# Determining Disease Intervention Strategies Using Spatially Resolved Simulations

**DOI:** 10.1371/journal.pone.0080506

**Published:** 2013-11-14

**Authors:** Mark Read, Paul S. Andrews, Jon Timmis, Richard A. Williams, Richard B. Greaves, Huiming Sheng, Mark Coles, Vipin Kumar

**Affiliations:** 1 Department of Electronics, the University of York, York, United Kingdom; 2 Department of Computer Science, the University of York, York, United Kingdom; 3 Centre for Immunology and Infection, Department of Biology and HYMS, University of York, York, United Kingdom; 4 Torrey Pines Institute for Molecular Studies, San Diego, California, United States of America; University of Catania, Italy

## Abstract

Predicting efficacy and optimal drug delivery strategies for small molecule and biological therapeutics is challenging due to the complex interactions between diverse cell types in different tissues that determine disease outcome. Here we present a new methodology to simulate inflammatory disease manifestation and test potential intervention strategies *in silico* using agent-based computational models. Simulations created using this methodology have explicit spatial and temporal representations, and capture the heterogeneous and stochastic cellular behaviours that lead to emergence of pathology or disease resolution. To demonstrate this methodology we have simulated the prototypic murine T cell-mediated autoimmune disease experimental autoimmune encephalomyelitis, a mouse model of multiple sclerosis. In the simulation immune cell dynamics, neuronal damage and tissue specific pathology emerge, closely resembling behaviour found in the murine model. Using the calibrated simulation we have analysed how changes in the timing and efficacy of T cell receptor signalling inhibition leads to either disease exacerbation or resolution. The technology described is a powerful new method to understand cellular behaviours in complex inflammatory disease, permits rational design of drug interventional strategies and has provided new insights into the role of TCR signalling in autoimmune disease progression.

## Introduction

The stochastic set of interactions that continually occur between different immune cells is essential for normal immune function but can also lead to formation of autoimmune pathology. Disease pathogenesis and progression is a result of complex cellular interactions spanning multiple spatial compartments, in which the role of cells and molecules is dynamic. Molecular- or cellular-level stochastic events occurring in a small collection of cells can dominantly affect disease outcome [1]. These processes are poorly understood as it is only possible to capture small windows of time and space using multiphoton confocal imaging or fixed time points using tissue sections and flow cytometric (non-spatial) analysis. Yet it is these complex set of behaviours at the single-cell level, in the context of time, space and location that lead to disease outcomes. A full capture of behaviours from the single cell to the organism level is key to understanding the pathways driving disease pathology, and using rational engineering principles in designing intervention strategies.

Agent-based simulation (ABS) is a technology that can be used to capture individual cellular behaviours *in silico*. Cells are explicitly captured as agents within a computational model permitting the spatial and temporal capture and analysis of heterogeneous and stochastic biological factors and events [2]. The rules governing how individual agents behave are designed to reflect interpretations of biology, however, numerical quantification of many key biological parameters are not possible and thus pose a challenge to the meaningful application of ABS to complex biological systems. Additionally, it is often unclear which aspects of a biological system are integral to disease, and then how they are best represented in a simulation that is by nature an abstraction of the biology. The method described addresses these issues by continually calibrating simulations against experimental data during simulation development, thereby guiding the capture of biological elements in simulation until they replicate the dynamics of the biological system. Crucial to simulation development is the mapping of biological rates and stochastic events into simulation parameters. These biological factors are often poorly characterized, or do not translate directly into simulation, yet can be highly influential in disease manifestation. We employ statistical analysis [3] of the simulations to determine where results may be assumed representative of the biology and experiments performed, or are rather the result of incomplete biological knowledge.

Simulations created through our method capture inflammatory disease at the resolution of individual heterogeneous cells, as opposed to capturing only population numbers, and reveal the role of their interactions and that of spatial compartments in disease. These simulations can highlight unique cellular interactions as well as potential novel targets for disease intervention. By perturbing the biological factors represented in simulation, such as cellular interactions, expressions of molecules, sensitivities, probabilities and durations of events, the roles of key factors in the induction of disease pathology are elucidated. Treatment strategies, regimens and doses can be simulated and explored, and their influence on disease outcome determined. 

In the present report we describe our method, and demonstrate it by investigating the onset and recovery of a T cell-mediated inflammatory disease. We construct ARTIMMUS, a simulation of the murine autoimmune disease experimental autoimmune encephalomyelitis (EAE) that spans multiple cellular and spatial compartments and where a clinical disease course precedes spontaneous recovery [4,5]. ARTIMMUS captures five spatial compartments, and seven distinct cell populations that have been shown to be critical in disease formation and resolution. We investigate the robust nature of the negative feedback pathway leading to spontaneous recovery, and how its disruption through either splenectomy or TCR-signalling inhibition can affect disease outcomes.

## Results

### Method for analysing inflammatory disease and intervention strategies through simulation

**Figure 1:**
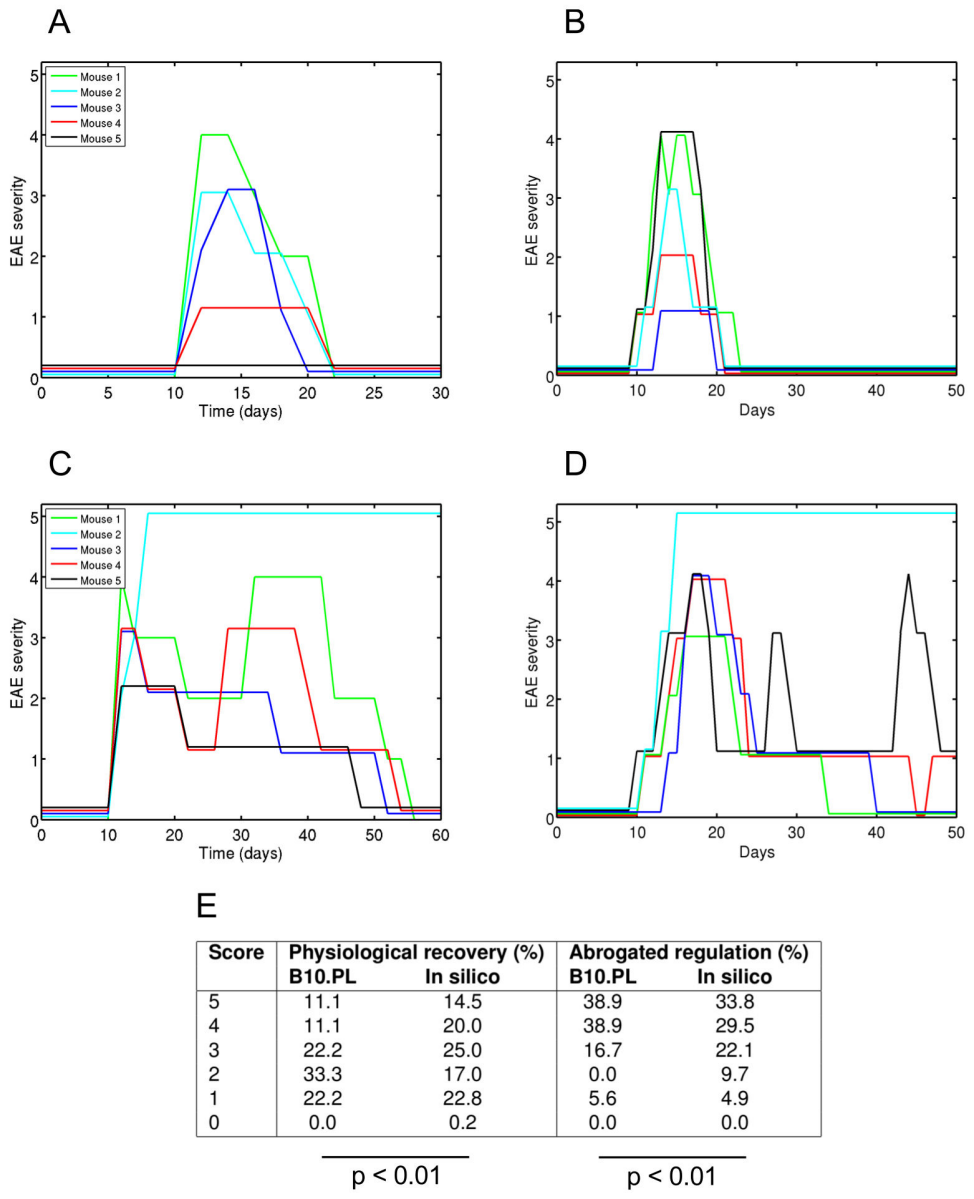
EAE dynamics in ARTIMMUS are consistent with those of B10.PL mice. Immunization for EAE leads to physiological recovery from autoimmunity in (A) ARTIMMUS and (B) B10.PL mice [31]. Abrogation of regulation delays recovery from EAE in (C) ARTIMMUS and (D) B10.PL mice [31]. 5 mice/simulations in each group. (E) The maximum scores in mice and ARTIMMUS simulations, as a percentage of the group, are statistically consistent. Data represents physiological recovery following induction of EAE, and laboured recovery following abrogation of regulatory pathway by which CD8Treg apoptose encephalitogenic CD4Th1 cells. *In* *vivo* data adapted from [8].

Our method produces a spatial- and cellular-resolved simulation which is demonstrated to appropriately capture the biological factors responsible for clinical disease, thus reproducing disease dynamics (Figure 1). Appropriate simulation representations of cells, the rules governing their behaviour, their interactions and spatial compartments are derived through explicit modelling of the biology which then forms the basis of the simulation (methods and materials). Explicit domain modelling helps highlight and resolve inconsistencies in the literature and presents a coherent view of the biology under investigation. It helps ensure that the simulation is of sound scientific grounding, and makes the assumptions and abstractions of the domain transparent [6]. Simulation development is incremental, driven by repeated calibration against *in vivo* behaviour. Simulation representations and abstractions of cells, molecules and spatial compartments are progressively refined until simulation dynamics qualitatively reflect the biology. Calibration of the simulation is performed against experimental data to ensure an appropriate representation and consistency with *in vivo* data (Figure 1). Calibration is performed against multiple experiments to avoid calibrating the simulation to a single data point in the space of experiments that can be performed, and to prevent over-fitting simulations to calibration data (methods and materials). 

We employ a statistical analysis to establish simulation robustness to unknown aspects of the biology [3,7] (table S1). The robustness analysis (methods and materials) establishes where simulation dynamics critically depend on particular parameters. These criticalities and the parameter values at which significant changes in simulation behaviour occur are examined to ensure they are reasonable and biologically plausible. The analysis reveals the extent to which simulation dynamics depend on biological factors that have not been well characterized, and this information helps to guard against misinterpreting simulation results and drawing unsupported conclusions. 

Experiments to investigate the role of cells and their interactions, and to simulate intervention strategies, are performed by identifying and manipulating the rules governing cell behaviour. Where an intervention blocks or stimulates a target, the simulation rules governing the behaviours resulting from interaction with that target are amended. Cells and compartments can be instantaneously added to or removed from the simulation. These *in silico* interventions can be applied at any time, and can be engineered free of the undesirable side-effects often associated with *in vivo* interventions, such as simultaneously affecting several cell populations.

### Establishing the role of immune pathways in inflammatory disease

We have used the ARTIMMUS simulation to examine the role of molecular-level pathways and components in inflammatory disease. Recovery from the clinical symptoms of EAE is mediated through the killing of encephalitogenic CD4^+^ Th1 (CD4Th1) cells by a coordinated effort of regulatory CD4^+^ (CD4Treg) and CD8^+^ T cells (CD8Treg) [4,5,8,9]. The effector CD4Th1 cells are only susceptible to regulation for the time following differentiation during which they express Qa-1:TCR-peptide complexes recognized by the CD8Treg [9,10]. We examined the probability that recognition of an activated CD4Th1 effector cell by an effector CD8Treg leads to the successful induction of apoptosis in the CD4Th1 cell (which we term “regulatory efficacy”, experimental procedure in methods and materials), and how this impacts disease manifestation and recovery.

**Figure 2:**
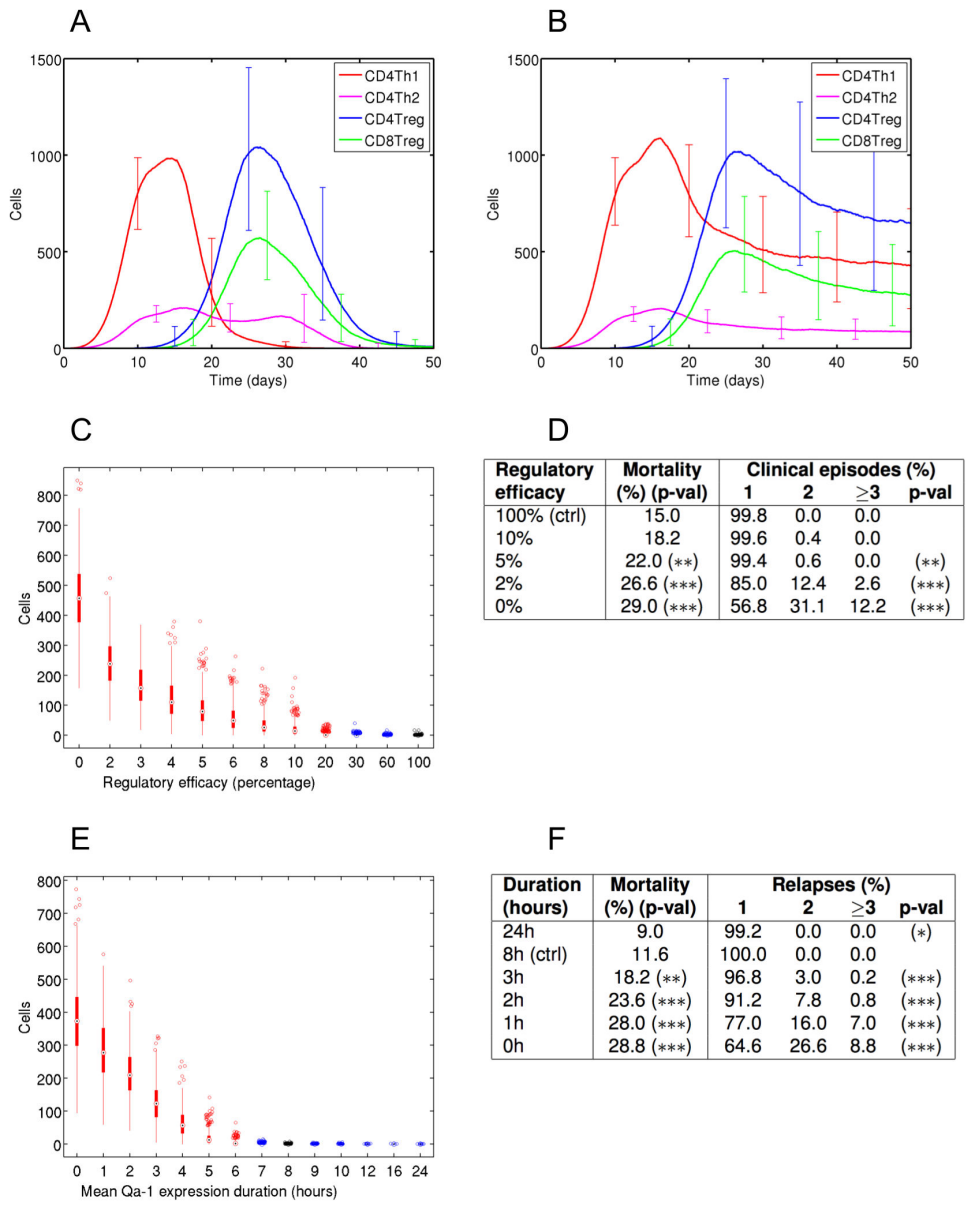
The role of regulatory pathways in EAE is elucidated through ARTIMMUS. (A & B) The effector T cell dynamics in ARTIMMUS: (A) recovery from EAE is mediated by CD8Treg killing of encephalitogenic CD4Th1 cells, which return to basal levels 35 days post-induction of EAE; (B) recovery is delayed and the encephalitogenic CD4Th1 cell population persists following complete abrogation of CD8Treg ability to apoptose them. Recovery from EAE is highly robust to disruption of this pathway. (C) Number of CD4Th1 cells present at 40 days, and (D) rates of mortality and clinical relapse when the probability that a CD8Treg successfully induces apoptosis in CD4Th1 target cells is reduced from 100% (control). CD4Th1 express Qa-1 for 8 hours following differentiation, during which time they are susceptible to CD8Treg killing. (E) Number of CD4Th1 present at 40 days and (F) rates of mortality and clinical relapse for different durations of Qa-1 expression. (C & E), black bars represent the control, red and blue indicate scientific significance and insignificance respectively. * = p<0.05; ** = p<0.01; *** = p<0.001; ‘ctrl’ indicates the control group.

We found that capacity for effective regulation is very robust to disruption of regulatory efficacy. In the control case regulatory efficacy is 100%: a CD8Treg binding with a target CD4Th1 cell kills the target cell with a probability of 100%, and the encephalitogenic CD4Th1 population is typically eradicated by 40 days post-induction for EAE (Figures 2A and 2C). A reduction in regulatory efficacy to 20% prompts a scientifically significant increase in the CD4Th1 population size at 40 days (Figure 2C), and an efficacy of just 3% halves the population size that results from complete absence of regulation (Figure 2C). Substantial reductions in regulatory efficacy are required to increase mortality rates (Figure 2D). In the control case 15% of simulations perish, a reduction from 100% to 5% efficacy significantly increases this to 22%, and complete absence of regulation incurs a 29% mortality rate. Substantial reductions in regulatory efficacy are also required to increase rates of clinical relapse (Figure 2D). Control simulations do not undergo clinical relapses, and reductions of regulatory efficacy to 5%, 2% and 0% incur relapses in 0.6%, 15% and 43% of simulations respectively. Substantial reductions in regulatory efficacy are required to increase the duration of clinical symptoms (Figures S1 and S2): the median duration of first clinical episode (excluding death) is 10 days in the control group, 11 days for an efficacy of 20%, 15 days for an efficacy of 2%, and 20 days for an efficacy of 0%. These results demonstrate the robust and redundant nature of CD8Treg regulation in EAE: very low regulatory efficacies remain effective in modulating CD4Th1 cell numbers, reducing the mortality rate and duration of clinical episodes, and preventing relapses.

Next we examined how the duration of Qa-1 expression by effector CD4Th1 cells impacts on disease pathology (experimental setup in methods and materials); encephalitogenic CD4Th1 cells are susceptible to apoptosis induction by CD8Treg only while expressing Qa-1:TCR-peptide complexes. We found that reducing the mean Qa-1 expression duration reveals qualitatively similar results to disruption of regulatory efficacy. A significant increase in CD4Th1 number at 40 days is incurred when the mean duration of Qa-1 expression is reduced from the 8 hour control to 6 hours (Figure 2E), and a duration of only 3 hours still halves the CD4Th1 population that otherwise results from 0 hours. Reducing the duration from 8 to 3 hours increases the mortality rate from 12% to 18%, and a reduction to 2 hours doubles it to 23% (Figure 2F). Large reductions in duration are required to prompt relapsing disease: the control group experiences only mono-phasic disease, the relapse rate is 3% for durations of 3h, 9% for 2h, 23% for 1h, and 35% for a mean duration of 0h (Figure 2F). We found little clinical benefit in increasing the Qa-1 expression duration: tripling duration time to 24h reduces mortality rate from 11.6% to 9%, a larger proportion of simulations experience maximum disease scores of 1 (as opposed to higher scores), and the median duration of clinical episodes amongst non-fatal simulations is reduced from 10 days to 9 (Figure S3).

Simulations developed through our method provide insight into how molecular-level events and pathways influence disease pathogenesis. Our results demonstrate a considerable redundancy in the ability of the CD8Treg population to effectively regulate the encephalitogenic CD4Th1 population. Our *in vivo* studies show CD4Th cells express Qa-1 between 4 and 24 hours post-stimulation (Figure S9). The present simulation results suggest that the bulk of regulatory interaction with CD8Treg occurs between 4 and 8 hours following differentiation of CD4Th1 into effector cells; simulated CD4Th1 cells express Qa-1 immediately following differentiation, when this terminates at 3 or less hours regulation is ineffective, and extension beyond 8 hours has little effect on recovery.

### Establishing the role of spatial compartments in disease

**Figure 3:**
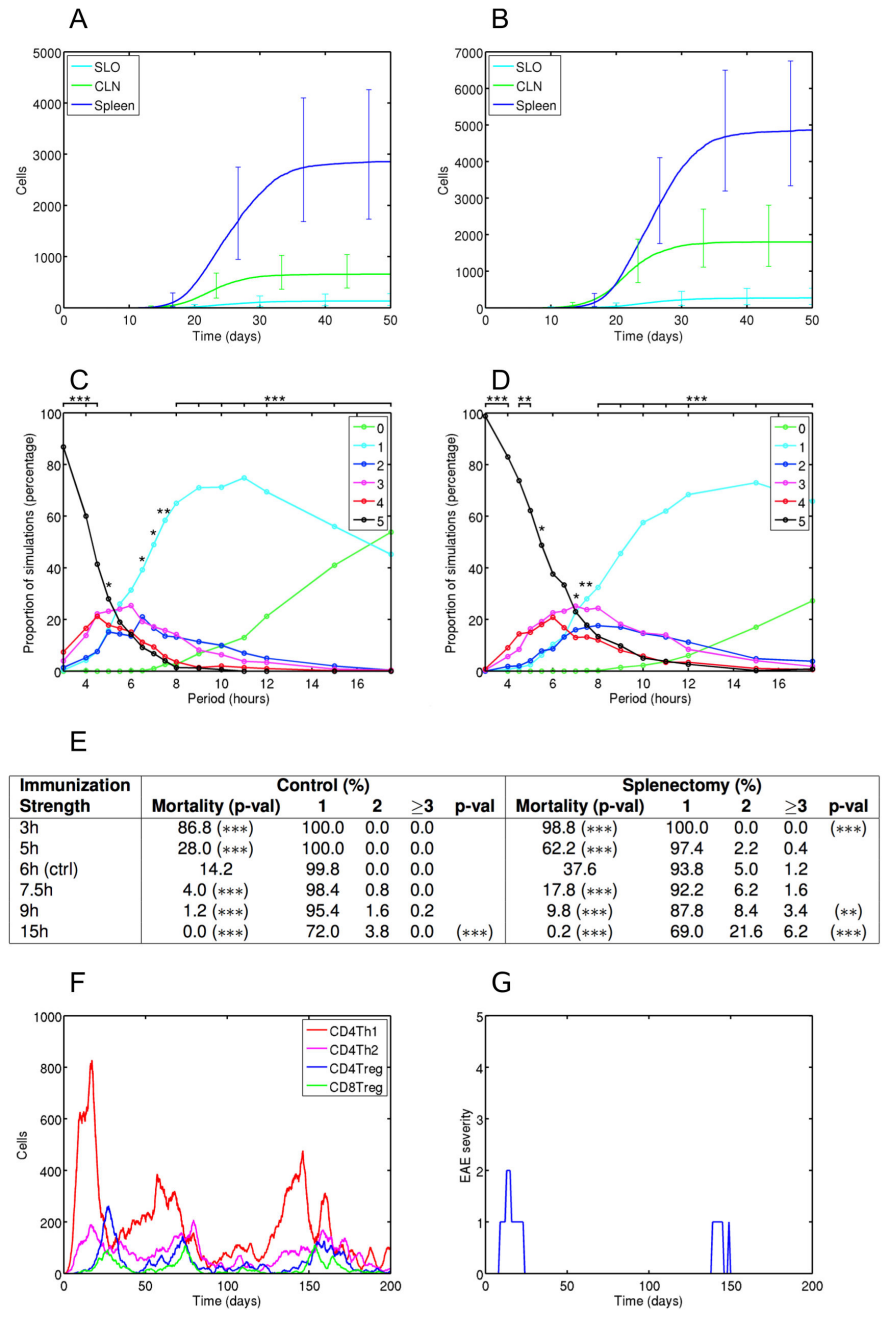
Splenectomy reduces Treg cell priming, and results in more severe clinical autoimmunity. The spleen is a major source of CD4Treg (A) and CD8Treg (B) priming; graphs show cumulative count of primed cells by compartment. We have investigated the effect of splenectomy for different strengths of immunization for EAE, represented as periodic placement of immunogenic MBP-presenting DCs in the SLO compartment: low periods represent stronger immunizations (see methods), 6h is the control. (C & D) The proportion of simulations that reach particular maximum EAE severities as a function of strength of immunization for EAE, for control (C) and splenectomy (D) groups. A-test effect magnitude levels are given: 1, 2 and 3 *'s represent small, medium and large effects respectively. (E) Mortality rates and incidence of clinical episodes over 200 days as a function of immunization strength, for control and splenectomy groups; *= p<0.05; ** = p<0.01; *** = p<0.001. Proportions of simulations contracting no clinical disease not shown. (F & G) Effector T cell dynamics for a single splenectomized simulation execution (F), and the corresponding clinical scores over time (G). An attenuated immunization for EAE is used (period = 12h). The reduced Treg populations resulting from splenectomy are unable to eradicate the encephalitogenic CD4Th1 population.

Our method develops spatially resolved simulations that can be used to investigate the role of specific spatial compartments or organs in disease pathology. We have contrasted *in silico* splenectomy and control experiments with *in vivo* data and found that the spleen is a primary source of regulatory T cells that expedite recovery from antigen-induced EAE (*in silico* splenectomy described in methods and materials). Our data are consistent with the earlier study demonstrating a role of the spleen in promoting recovery from EAE: splenectomy in rats prior to the induction of EAE increases the disease severity with poor recovery as reflected by higher mortality rate and chronic disease [11]. Accordingly, our results reveal the spleen as a major site of Treg priming (Figures 3A and 3B), and that splenectomy significantly reduces the CD4Treg and CD8Treg population sizes (Figures S4A, S4B and S4C). We investigated the diminished recovery from autoimmunity following splenectomy as a function of strength of immunization for EAE. Splenectomized groups experience higher clinical disease scores than control groups across all immunization strengths (Figures 3C and 3D). In contrast with controls, splenectomized simulations suffer a higher mortality rate and have a greater tendency towards relapsing clinical disease, especially for weaker immunizations (Figure 3E). The reduced Treg populations that result from splenectomy are not always able to completely abrogate encephalitogenic CD4Th1 populations. This results in their re-expansion (Figure 3F) and relapses of clinical autoimmunity (Figure 3G).

### Characterizing interventional strategy and its operation

We employed ARTIMMUS to explore a potential intervention strategy, predicting treatment effectiveness and providing an understanding of treatment effect on cell populations. As a T cell-mediated autoimmune disease, EAE is potentially treatable using CD3 (2C11) antibodies [12-14]. Our *in vivo* studies have also revealed that anti-CD3 Ab can protect mice from an antigen-induced model of EAE (Figure S10). We asked whether a hypothetical anti-CD3 non-mitogenic FcR-non-binding non-depleting antibody intervention that blocks all T cells, including Treg, can protect from EAE. Various efficacies of this hypothetical Ab are simulated in ARTIMMUS (experimental procedure in materials and methods). Administrations at days 4 or 15 have been simulated, to correspond with onset of encephalitogenic T cell expansion and peak clinical symptoms respectively.

**Figure 4:**
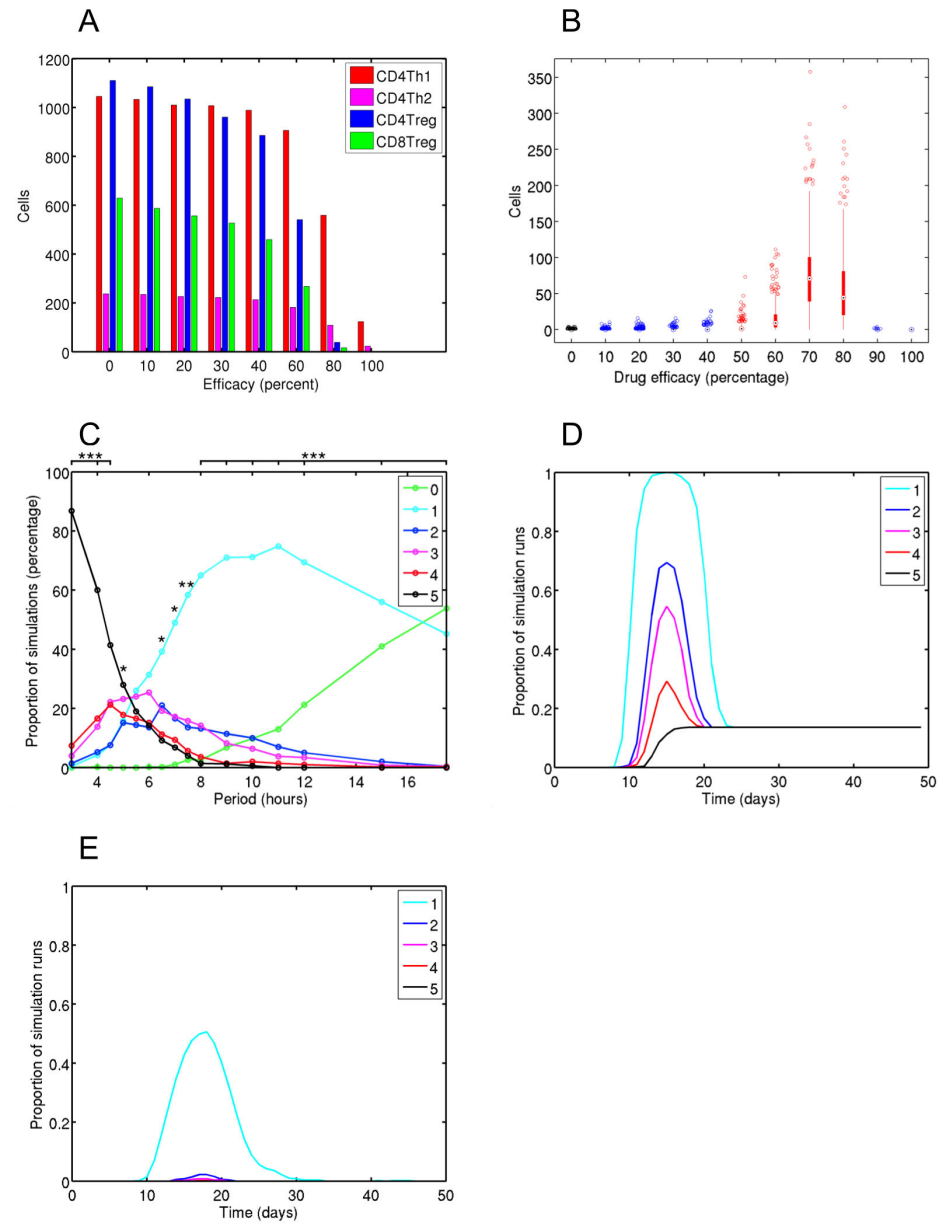
Effector T cell and clinical disease dynamics given anti-CD3 intervention at day 4. Various efficacies of anti-CD3 intervention have been administered at day 4, which corresponds with encephalitogenic T cell priming. (A) Median effector T cell peak population sizes. (B) CD4Th1 population sizes at 40 days post-induction of EAE; red and blue bars indicate large and non-large effect magnitude changes with respect to the control group, in black. (C) Proportion of simulations that reach a particular maximum clinical disease score. A-test effect magnitude levels are given: 1, 2 and 3 *'s represent small, medium and large effects respectively. (D & E) Proportion of simulations contracting particular clinical scores or greater over time, for control (D) and a drug efficacy of 80% (E).

When administered at day 4, all efficacies of anti-CD3 Ab reduce the sizes of all T cell population expansions (Figures 4A and S5). CD8Treg and CD4Treg populations are more sensitive to the intervention than CD4Th2 and CD4Th1 cells, showing greater effect magnitudes of reduction given the same efficacy (Figure S6A). Despite reducing peak T cell numbers, increasing intervention efficacies between 0 and 70% prolongs both autoimmune and regulatory T cell responses, and increases the number of effector CD4Th1 cells at 40 days (Figures 4B and S5). At efficacies above 70% these trends reverse: the number of CD4Th1 remaining at 40 days decreases (Figure 4B), as does the duration of both autoimmune and regulatory responses (Figure S5), and the maximum clinical scores attained reduces (Figure 4C). Increasing intervention efficacies reduced the maximum disease severities experienced: the proportions of simulations scoring 2-5 decreased, and the proportions scoring 1 or 0 increased (Figure 4C). Whereas all control simulations (0% efficacy) experience clinical symptoms, an 80% intervention efficacy protects 40% from clinical disease (Figures 4E and S6B). Of the remaining 60% the majority experience only grade 1 symptoms, but disease persists amongst this group to day 30 in contrast with day 23 in the control group (Figures 4D and 4E), and 8% experience clinical relapses in contrast to 0% in the control group (Figure S6B).

**Figure 5:**
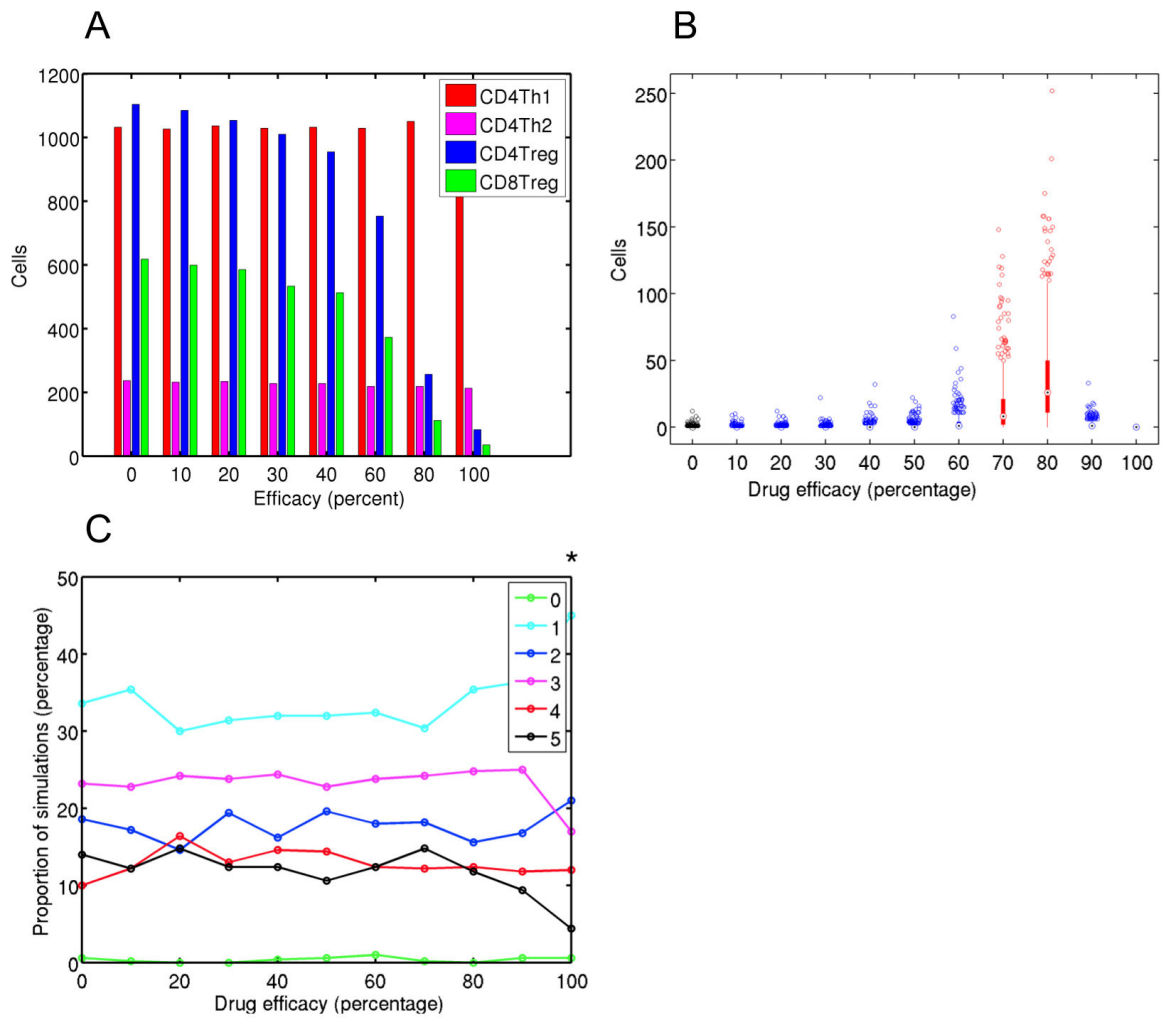
Encephalitogenic T cell and clinical disease dynamics resulting from anti-CD3 intervention at day 15. Various efficacies of anti-CD3 intervention have been administered at day 15, which follows the establishment of clinical autoimmunity. (A) Median effector T cell peak population sizes. (B) Box plot showing CD4Th1 population sizes at 40 days post-induction for EAE for various drug efficacies. Red and Blue bars indicate scientifically significant and non-significant changes with respect to the control group, in black. (C) Proportion of simulations that reach a particular maximum clinical disease score for various anti-CD3 efficacies. Vargha-Delaney A-test effect magnitude levels are given: 1, 2 and 3 *'s represent small, medium and large effects respectively.

We examined intervention at day 15, following the establishment of peak clinical autoimmunity. Treg population sizes reduce with increasing efficacy, but peak CD4Th1 and CD4Th2 numbers are unaffected (Figures 5A and S7). A significant increase in day 40 CD4Th1 population size is found for efficacies of 70% and 80% (Figure 5B). In terms of clinical disease, an efficacy of 100% has a small effect in reducing maximum disease scores attained, and no other efficacies revealed an effect (Figure 5C). However, high efficacies up to 80% prolong the period over which clinical symptoms present (Figure S8), though not to the same degree as found for administration at day 4 (Figure 4E). These results show that intervention following the establishment of clinical autoimmunity can delay physiological recovery from disease. 

It is clear from our results that anti-CD3 Ab treatment is not uniformly beneficial in treating EAE. Only for efficacies greater than 80%, administered at either day 4 or 15, is the drug effective in reducing maximum clinical scores, reducing the duration of clinical episodes, and preventing clinical relapses. This may be explained through the suppressive action of anti-CD3 not only on encephalitogenic T cell populations, but also on the Treg populations that regulate them. Tregs are more sensitive to anti-CD3 treatment, since its effect is twice felt: a reduced number of encephalitogenic T cells are primed, phagocytosed and presented for Treg priming; and Treg ability to bind APCs is reduced through direct action of the treatment.

## Discussion

We have presented a method for elucidating the cellular and spatial basis of systemic inflammatory disease, and investigating potential intervention strategies, through computational agent-based simulation. Through the explicit capture of heterogeneous cell populations and spatial compartments, the implications of cellular-level events, behaviours and interventions on disease are exposed. The method has been demonstrated by investigating the cellular and spatial factors principally responsible for the onset and recovery of autoimmunity in EAE, a T-cell-mediated murine prototype for multiple sclerosis, and the potential for a hypothetical intervention strategy to alleviate disease. 

The computational spatial- and cellular-resolved simulations our method develops are highly flexible in permitting examination and analysis of any aspect of disease, including individual cell-, population-, compartment- and disease-level phenomena. The same flexibility characterizes the interventions that may be engineered into a simulation to understand the mechanistic foundations of its dynamics. Any aspect of cellular or intervention behaviour can be manipulated, free of any side effects present *in vivo*. This constitutes a powerful means to understand and reason about disease manifestation and recovery. We have demonstrated this by employing simulation in characterizing the considerable redundancy in the ability of the CD8^+^ regulatory T (CD8Treg) cell population to effectively regulate encephalitogenic T cells: the clinical and cellular implications of altering CD8Treg regulatory capacity by single percentage points were revealed. Our data are consistent with the *in vivo* studies using CD8-depletion or Qa-1-deficient animals [15-17]. Furthermore, it is consistent with the resistance of the wild-type but not Qa1-deficient B6 mice to the re-induction of disease through immunization with antigen emulsified in complete Freund’s adjuvant [18]. We find that a persisting or relapsing disease pathology requires near abolishment of the capacity for CD8Treg to apoptose encephalitogenic T cells. SJL mice are highly susceptible to EAE and prone to relapses, both induced and spontaneous, following initial induction of disease [19]. Our data suggest that substantial dysfunction in T cell-mediated specific regulation can lead to such disease pathology. 

We have also demonstrated how the spatial underpinnings of disease can be elucidated. We have identified the spleen as a dominant site of regulatory T cell (Treg) priming, and highlighted how the reduced Treg populations that result from splenectomy are not always able to completely abrogate encephalitogenic T cell populations which can lead to a remitting-relapsing disease pathology. Our results are consistent with *in vivo* studies showing that CD4Treg cells are primed in the spleen [20] and cervical lymph nodes [21] of mice following the induction of EAE, and can explain why splenectomy of rats induced into EAE worsens their clinical outcome [11]. Our simulation was not directly informed by, or calibrated against, the rat model of EAE, and the consistency of our results with those of Ben-Nun et al. [11] demonstrates the generality of our method in exploring disease. Simulations created through our method are versatile, allowing diverse aspects of disease to be explored. We have previously used the present simulator to examine possible roles that CD200 negative signalling of dendritic cells (DCs) has on their priming of T cell responses [22], whether recovery in EAE requires regulatory and encephalitogenic T cells to be co-primed on the same DCs, and the spatial and temporal characteristics of DC licensing in cytotoxic T cell responses [23].

Our method allows for the simulation of potential intervention strategies, and can be used to predict treatment effectiveness and reveal the mechanistic basis of treatment on cellular or molecular components. We have investigated the potential for a hypothetical anti-CD3 non-mitogenic FcR-non-binding non-depleting antibody intervention that inhibits T cell TCR interaction to expedite recovery from autoimmunity. Our results show that this anti-CD3 Ab intervention is not uniformly beneficial in treating EAE, and that very high efficacies (which can translate into doses) are required to alleviate clinical autoimmunity. These results are consistent with clinical trials of anti-CD3 intervention in new-onset type 1 diabetes. Large cumulative doses were required for maintenance of beta-cell function [24,25], whereas phase III trials using low doses failed to meet their primary end points [25,26]. As with our present results in simulating EAE, treatment during early development of diabetes has proven more effective [27]. Our results highlight the importance of considering the effect of treatments not only on target T cell populations, but on regulatory pathways of the immune system that modulate them. Although our simulated anti-CD3 intervention is not pharmacologically identical to those employed in clinical trials in type 1 diabetes, our results are nevertheless consistent with the finding that all but the highest efficacies of intervention are shown to be beneficial. These results demonstrate how *in silico* experiments following our method facilitate rational design of drug intervention strategies. Simulating hypothetical drug designs can inform the drug discovery process: our present results suggest that any intervention that non-specifically blocks all TCR interactions is only effective in treating EAE at very high efficacies. Furthermore, *in silico* experiments that accurately replicate the pharmacology of an existing drug could inform the design of clinical trials. 

The principal challenge in exploring complex biological systems that are not completely understood through simulation is that this same lack of understanding can complicate simulation design. Simulations are highly abstract representations that make many assumptions of the biology. Computational biology publications rarely acknowledge this: assumptions are rarely made clear, and their implications in simulation results rarely appreciated. Our unique method provides for the first time a principled approach to studying disease through simulation, where the capture of the biology and assumptions made is informed by evidence of improved capture of *in vivo* behaviours. Completed simulations are statistically analysed to determine and expose the reliance of simulation behaviours on aspects of the biology that are poorly characterized; this novel analysis guards against drawing ill-supported biological conclusions from simulation-based experimentation. Our method provides evidence and confidence that simulation is an appropriate capture of the biology. These technologies pave the way for using simulation in the design of clinical trials, or providing personalized medicine, where confidence in a simulation’s predictive accuracy is essential. Finally, it will be crucial to developing simulations that can help determine a particular individual’s immune response or clinical symptoms to particular treatment strategies, given their unique personal medical history, immunological constitution and current medical circumstances.

## Materials and Methods

### Ethics Statement

*In vivo* murine experiments (figures S9 and S10) were performed in compliance with the Federal and Institutional guidelines and have been approved by the Institutional Animal Care and Use Committee of the Torrey Pines Institute for Molecular Studies, San Diego, CA, USA.

### ARTIMMUS, an EAE simulator

The ARTIMMUS (artificial murine multiple sclerosis) simulation was implemented in Java and makes use of the MASON simulation framework [28] (Figure S11A). It explicitly captures 7 cell populations: CD4Th1 cells, CD4Th2 cells, CD4Treg cells, CD8Treg cells, microglia, dendritic cells (DCs) and neurons. 

5 spatial compartments are explicitly represented: the central nervous system; a cervical lymph node; the circulatory system; the spleen; and a generic lymph node named the secondary lymphoid organ (SLO). ARTIMMUS employs a 2-dimensional lattice-grid based spatial representation for each compartment, and these compartments are networked allowing cells leaving one compartment to enter another. The network, and migration of cells between compartments are shown in Figure S11B. Cells are represented as explicit entities that occupy and move between grid-spaces on the lattice grids. Several abstractions of cytokines and soluble factors are represented, with abstractions are made by function: generic ‘type 1’ and ‘type 2’ cytokines, and a ‘demyelinating agent’ are represented as concentrations within each grid-space. All are subject to decay and diffusion.

Immunization for EAE is captured through the insertion of type-1 polarized MHC-II:MBP expressing immunogenic DCs (immunization DCs) into the SLO compartment. An initial number of immunization DCs are placed in the SLO at time of EAE induction, and a linearly decreasing number are added periodically for some time thereafter.

Cells in ARTIMMUS express particular receptors, such as co-stimulatory molecules and MHC:peptide complexes. When cells with corresponding molecules occupy either the same or adjacent grid-spaces, and if the required receptors are currently being expressed, then a binding between the two cells is made and this constitutes cellular signalling pathways that alter a cell’s state. In the case of T cells, the establishment of these bindings is probabilistic, to reflect T cell specificity. 

Many changes in cellular state are temporal in nature, occurring after some passage of time. These durations are not identical for all individuals of a particular cell type; cell-specific durations are drawn from a normal distribution, with simulation parameters describing the mean and standard deviation. Durations until a state transition occurs are drawn as required, and performed when the corresponding time has elapsed.

The ARTIMMUS simulation, full details of its implementation and default parameter values, is available for free download from http://www.ycil.org.uk/software-2.

### Domain modelling

The domain model comprises three levels of abstraction, capturing cell- and molecular-level dynamics (level 3), the culmination of these dynamics into system-level events (level 2), and how these system level behaviours translate into phenomenon at the organism-level such as recovery from autoimmunity (level 1). No formal modelling syntax or semantics are employed in level 1, it is a highly abstract overview of the system being modelled and scopes the *in silico* work in terms of the biology. Level 2 is expressed using UML activity diagrams [29]. It is impossible to coherently model the entire cellular basis of disease pathogenesis and recovery on a single diagram, and such level 2 decomposes disease into various perspectives that are modelled individually. Each level 3 diagram focuses purely on one cell or molecule, and uses UML state machine diagrams [29] to describe their states and transitions between them. Examples of each level of modelling are shown in Figures S12, S13 and S14.

Details of simulation implementation are excluded from the domain model, and only the level 3 dynamics are used in specification of simulation code. This ensures that system-level behaviours emerge from the mass-integration of cell-level behaviours, rather than being directly encoded into the simulation. 

The full domain model for ARTIMMUS is available for download at http://www.ycil.org.uk/software-2.

### Calibration

The abstraction of EAE biology in ARTIMMUS has been informed throughout simulation design by systematically calibrating against *in vivo* experiments. The inability to replicate the dynamics of these experiments in ARTIMMUS, where this occurred, indicated that the biological abstraction was not correct, and thus motivated further development of ARTIMMUS's biological abstraction. 

ARTIMMUS was calibrated against two experiments. These are the physiological recovery of mice induced into EAE (as reported in [30,31]), and laboured recovery following abrogation of the CD4Treg and CD8Treg regulatory pathway (two such experiments have been performed *in vivo*: anti-TCR antibodies that depleted CD4Treg populations for 3 weeks [31], and perforin knock-out mice were used in which CD8Treg are unable to apoptose encephalitogenic T cells [8]). ARTIMMUS abstractions and parameters were calibrated against the specifications for T cell dynamics as supplied by VK for both experiments. 

Once ARTIMMUS abstractions and parameters were finalized a metric for grading simulation executions in terms of the 5-point disease severity scale employed *in vivo* was devised and calibrated. The ARTIMMUS abstraction for demyelination in the central nervous system is neuronal death; neurons are killed (the abstraction of demyelination) by sufficient concentrations of soluble demyelinating agent and are replaced thereafter. *In silico* EAE grades are based on rates of neuronal death. Time-series data concerning the rates of neuronal death are smoothed with a sliding window filter, and EAE scores are assigned by thresholding the rates of neuronal death. The sliding window size and threshold values are obtained through independent calibration against the two experimental setups used above: physiological recovery following induction of EAE, and laboured recovery following abrogation of the regulatory pathway. Threshold values were calibrated to: 1) reflect the frequency at which EAE scores change *in vivo*; 2) reflect the proportion of mice that reach particular maximum EAE scores; and 3) minimise the difference between thresholds acquired through calibration against each experiment. Optimal sliding window sizes and thresholds were obtained by minimising sum of squared normalized difference measures reflecting the preceding requirements. The *in vivo* 5-point disease severity scale is as follows [20]: 1, flaccid tail; 2, hind limb weakness; 3, hind limb paralysis; 4, whole body paralysis; 5, death.

### Robustness analysis

The robustness analysis is a one at a time analysis; the parameter under investigation is perturbed, whilst all others retain their default calibrated values. The effects of perturbation on various aspects of simulation behaviour (termed ‘responses’) are analysed, with 500 simulation executions performed for each perturbation. The range of perturbation is parameter-specific; if possible the full range of values are investigated, but for unbounded ranges perturbations of “reasonably” large magnitude are used, which can include several orders of magnitude. Hence, the range of values that a parameter may hold around its default value before a significant deviation in simulation behaviour occurs is ascertained. Significant deviations are defined as large effects as measured through the A-test, or ±1.0 in mean EAE score. The distance between the boundary at which significant deviations in simulation behaviour occur and the default parameter value is expressed as a percentage of the default value (termed an “index”). Where no boundary exists ‘Not a Number’ is assigned to the corresponding index. The smaller of the two indexes is termed the “robustness index”. Parameters are ranked for each response in order of increasing robustness indexes. The ranks for each parameter across all responses are summed and used to order a global robustness analysis table (Table S1). This table exposes those parameters to which simulation behaviour is particularly sensitive. The boundaries for parameters are examined to ensure that no unreasonable criticalities concerning parameter values exist; such occurrences suggest an inappropriate capture of some aspect of the biology in the simulation. Figure S15 demonstrates the analysis on a single parameter. The full robustness analysis of ARTIMMUS is available for download from http://www.ycil.org.uk/software-2. No unreasonable criticalities have been identified in ARTIMMUS.

### Statistics

Unless otherwise stated, all experimental groups comprise 500 simulation executions. Error bars on graphs indicate the inter-quartile range, and on such graphs the median values across all simulations are plotted.

The Vargha-Delaney ‘A-test’ [32], a non-parametric effect magnitude test, is used to establish magnitude of effect (also referred to as ‘scientific significance’) between control and experimental groups. This is used as the effect magnitude calculated is independent of the number of samples taken. The A-test contrasts populations A and B, and returns the probability that a randomly selected sample from population A is greater than a randomly selected sample from population B. Vargha and Delaney state that A-test scores of ≤0.44 or ≥0.56 indicate a small effect, ≤0.36 or ≥0.64 indicate a medium effect, and large effects are indicated by scores ≤0.29 or ≥0.71. 1, 2 and 3 *'s on graphs and tables represent small, medium and large effects respectively. The A-test is applied to data concerning the proportion of simulations experiencing particular maximum disease scores, differences in cell population expansion peaks, and CD4Th1 cells at 40 days.

The Mann-Whitney U test is performed to ascertain the statistical significance between control and experimental groups. This metric is often reported in this paper in reference to percentages of simulations that experience a particular numbers of clinical episodes, however it is applied to the raw data not the percentages themselves. For example, to contrast a group where 100% of simulations experienced a single clinical episode and another where 80% experienced one clinical episode and 20% experienced two, the U test is input with one population of 500 samples where each sample holds the value 1 and another population where 400 samples hold the value 1 and the remaining 100 hold the value 2. Fisher's exact test is used to ascertain the statistical significance of changes in mortality rates between control and experimental groups. 

The following indicators of significance level are used for the U test and Fisher's exact test: * = p<0.05; ** = p<0.01; *** = p<0.001. All statistical tests are conducted using Matlab implementations. The A-test is calculated using the following Matlab code:

 function A = Atest(X, Y)

 [p,h,st] = ranksum(X,Y,’alpha’,0.05);

 N = size(X,1); M = size(Y,1);

 A = (st.ranksum/N - (N+1)/2)/M;

### Efficacy of regulation

A CD8Treg cell can apoptose a Qa-1:CDR1/2 expressing effector CD4Th1 cell only if the two cells occupy the same or adjacent locations in the spatial grid. The CD8Treg must establish a TCR:Qa-1 binding with the CD4Th1, and this probabilistic to represent non-perfect specificity of T cells. If a binding between the cells is established, the CD8Treg apoptoses the CD4Th1 subject to some probability, which is represented by a single parameter, representing ‘regulatory efficacy’. This probability is the same for all CD8Treg cells, and is varied between 0% and 100% in the experiments reported.

A CD4Th cell expresses Qa-1:CDR1/2 for period of time following its differentiation into an effector cell. The duration is established independently for each cell and is drawn from a normal distribution with a mean of 8 hours and a standard deviation of 1 hour. The mean of this distribution is varied between 0 and 24 hours in the experiments reported.

### Splenectomy

Splenectomy is implemented in ARTIMMUS by substituting the spleen spatial compartment with an alternative “splenectomy-spleen”. This alternative is networked with other spatial compartments to facilitate inter-compartment cellular migration as normal, but it has zero capacity: any cell entering the splenectomy-spleen immediately exits. The splenic dendritic cells that reside in the ordinary ARTIMMUS spleen do not exist in the splenectomy-spleen. *In silico* splenectomy is performed by substituting the splenectomy-spleen prior to simulation execution, which corresponds with performing an *in vivo* splenectomy prior to the induction of EAE.

### Anti-CD3 intervention

Anti-CD3 intervention reduces the probability that a T cell’s TCR successfully binds with a target MHC:peptide complex when the corresponding cells occupy the same or adjacent spatial grids. This interaction is already probabilistic to reflect the differing specificities of T cells, and as such this intervention further reduces the probability of successfully establishing bindings by the corresponding amount. The intervention is administered at either day 4 or 15, it affects all T cells in ARTIMMUS equally, its effect is immediate from time of administration and it persists for the duration of the simulation. 

An efficacy of 0% corresponds to the control case of no intervention. A 100% efficacy prevents all T cell TCR-bindings from time of administration onwards. Although this implementation has no explicit notion of intervention dose, “efficacy” may be thought of as a combination of dose and the binding strength of the antibody with its target.

## Supporting Information

Figure S1 **Lower regulatory efficacies increase duration of clinical episodes, and raise the mortality rate.** The proportion of simulations contracting particular durations of clinical episodes or symptoms, for regulatory efficacies of 100% (A), 20% (B), 5% (C), 2% (D), 0% (E). Where applicable, data for clinical relapses are also shown. Where simulations perish, the duration of the clinical episode is from onset of symptoms until end of observation at 50 days, hence the curves plateau at the death rate, and rise to a proportion of 1 at the end.
(TIF)

Figure S2 **Lower regulatory efficacies increase the severity of clinical episodes.** The proportions of simulations experiencing at least each level of severity of EAE over time, for regulatory efficacies of 60% (A), 5% (B), 3% (C), 2% (D), and 0% (E).
(TIF)

Figure S3 **Increasing the duration of Qa-1:peptide expression beyond 8 hours has a marginal effect on clinical symptoms.** The effect of altering mean duration of Qa-1:peptide complexes expression by effector CD4Th1 cells on the dynamics of clinical autoimmunity. (A) The proportions of simulations experiencing particular maximum clinical scores at any point in time. A-test effect magnitude levels are given: 1, 2 and 3 *'s represent small, medium and large effects respectively. (B & C) The proportions of simulations experiencing particular clinical scores or greater over time, for Qa-1:peptide complex expression durations of 8 hours (B) and 24 hours (C).
A cumulative distribution plot showing the proportions of simulations that experience particular durations of clinical symptoms or less for Qa-1 peptide complex expression durations of 8 hours (D) and 24 hours (E). Increasing the mean period of time for which encephalitogenic CD4Th1 cells express Qa-1:peptide complexes, necessary for their regulation by CD8Treg cells, marginally reduces the severity of EAE contracted and reduces the median duration of clinical symptoms amongst simulations that do not perish from 10 days to 9.
(TIF)

Figure S4 **The effect of splenectomy on recovery from EAE *in vivo*, and on Treg priming *in silico*.** (A & B) effector T cell dynamics in control (A) and splenectomy (B) groups. (C) statistical magnitudes effect of splenectomy on effector T cell population dynamics. The peak number of cells attained, and the times at which these occur, in each of 500 splemenctomized simulations are contrasted with similar data from a control group using the A-test. A-test effect magnitude levels are given: 1, 2 and 3 *'s represent small, medium and large effects respectively.
(TIF)

Figure S5 **Anti-CD3 intervention at day 4 suppresses all T cell population expansions.** Effector T cell population sizes over time, for anti-CD3 treatment efficacies of 0% (A), 60% (B), 70% (C), 80% (D), 90% (E), 100% (F). Higher intervention efficacies reduce effector T cell peak population sizes, but for efficacies under 80% encephalitogenic CD4Th1 cells persist for longer.
(TIF)

Figure S6 **Increasing day 4 anti-CD3 intervention efficacies reduces severity of EAE and T cell population expansions.** (A) Vargha-Delaney A-test scores indicating the effect magnitude of reduction in peak cell population for T cell populations under various intervention efficacies. 1, 2 and 3 *'s represent small, medium and large effects respectively. (B) Mortality rate and proportion of simulations experiencing relapses of clinical symptoms for various efficacies of anti-CD3 administered at day 4 post-induction for EAE. * = p<0.05; ** = p<0.01; *** = p<0.001.
(TIF)

Figure S7 **Effector T cell dynamics for various day 15 anti-CD3 intervention efficacies.** Effector T cell population sizes over time, efficacies of 0% (A), 70% (B), 80% (C), 90% (D), 100% (E).
(TIF)

Figure S8 **Increasing day 15 anti-CD3 efficacies reduce duration and severity of clinical episodes.** Proportion of simulations experiencing particular clinical scores or greater over time, for anti-CD3 efficacies of 0% (A), 70% (B), 80% (C), 90% (D), 100% (E).
(TIF)

Figure S9 **Dynamic expression of Qa-1 on T cells following their activation *in vivo*.** Groups of C57BL/6 mice were administrated intraperitoneally with a single dose of T cell activating anti-CD3 (200 μg, 2C11) antibody. Splenocytes were harvested at indicated time points 1h, 2h, 4h, 6h, 12h, 24h, 72h, 120 h, stained with anti-TCR, anti-CD4 and anti-Qa-1b, and subjected to flow cytometry. (A) Histogram data and (B) a bar graph showing percentage of cells stained from different animals. This data is one representative of at least three individual experiments.
(TIF)

Figure S10 **Anti-CD3 treatment prevents antigen-induced EAE *in vivo*.** (A) Groups of C57BL/6 female mice were one administered with 200 micrograms of anti-CD3 (2C11) antibody 5 days prior to the induction of disease. EAE was induced by immunization with MOG35-55 peptide emulsified in the Complete Freund's Adjuvant followed by 150 ng of pertussis toxin injection. Mice in the control group were administered with PBS. These data are representative of 2 independent experiments.
(TIF)

Figure S11 **The ARTIMMUS simulation.** (A) Screen shot of the simulation. Cells are coloured as follows: blue, non-effector CD4Th cells; red, effector CD4Th1 cells; white, effector CD4Th2 cells; yellow, CD4Treg cells; green, CD8Treg cells; purple, dendritic cells; peach, microglia; grey, neurons. (B) The spatial compartments represented in ARTIMMUS, and which cells are able to migrate between them.
(TIF)

Figure S12 **Example of level 1 domain modelling: culmination of organism-level phenomenon.** An abstraction depiction of the cells and their interactions believed to be responsible for clinical disease and subsequent recovery. This diagram scopes the *in* *silico* work, for example “protection against subsequent attempts to induce autoimmunity against CNS” is observed in clinical animals, but is beyond the scope of the present modelling work.
(TIF)

Figure S13 **Example of level 2 domain modelling: system-level events.** Immunization for EAE leads to neuronal apoptosis, expressed as a UML activity diagram.
(TIF)

Figure S14 **Example of level 3 domain modelling: cellular- and molecular-level dynamics.** The cellular dynamics of an encephalitogenic CD4Th cell, expressed as a UML state machine diagram.
(TIF)

Figure S15 **Example robustness analysis of the *TCell_AICDMean* parameter.** This parameter dictates the mean lifespan of effector T cells before they apoptose due to activation induced cell death (AICD). The analysis establishes the range of values that this parameter may take before significant deviations in various aspects of simulation behaviour take place. These aspects, termed responses, are as follows: the …Max and …MaxTime responses indicate the peak population size for each T cell population and the times at which this peak occurred. CD4Th1@40d represents the CD4Th1 population size at 40 days. Max EAE and EAE@40 represent the disease severity score at its peak and at 40 days; these measures are tested for significant deviation through both the A-test and ±1.0 of the default value. (A) A-test scores indicating how changes parametric perturbation influences simulation responses, the ‘large’ effect magnitude boundaries are indicated. (B) Change in EAE scores under parametric perturbation, ±1.0 boundaries are indicated. (C) Summary of robustness indices, lower and upper boundaries and indices for all response. RI, robustness index; LI, lower index; UI, upper index; LB, lower boundary; UB, upper boundary. For clarity NaN (not a number) is indicated by a period.
(TIF)

Table S1 **Summary of parameter robustness indexes, ordered by total rank.** Responses are indicated as follows: 1M, *CD4Th1* *Max*; 1MT, *CD4Th1* *Max* *Time*; 2M, *CD4Th2* *Max*; 2MT, *CD4Th2* *Max* *Time*; 4M, *CD4Treg* *Max*; 4MT, *CD4Treg* *Max* *Time*; 8M, *CD8Treg* *Max*; 8MT, *CD8Treg* *Max* *Time*; Th40, *CD4Th1* *at 40* *Days*; MEA, *Max* *EAE*; E40A, *EAE* *at 40* *Days*. Significant deviation is indicated through the A-test. ME and E40 represent *Max* *EAE* and *EAE* *at 40* *Days*, with significant deviations in response behaviour defined as a change of at least ±1.0 in the mean EAE score. Not-a-number values, representing no significant deviation in behaviour, are marked with a period for clarity. Response columns show the smaller of the two robustness indexes for each parameter-response combination. The ‘total’ is the sum of ranks for each parameter across all responses, with small response indexes being ranked highest.
(TIF)
