# Correction: Determining Disease Intervention Strategies Using Spatially Resolved Simulations

**DOI:** 10.1371/journal.pone.0128170

**Published:** 2015-05-08

**Authors:** Mark Read, Paul S. Andrews, Jon Timmis, Richard A. Williams, Richard B. Greaves, Huiming Sheng, Mark Coles, Vipin Kumar

The image for Fig 4C is incorrect. Please see the complete, corrected Fig 4 here.

**Fig 4:**
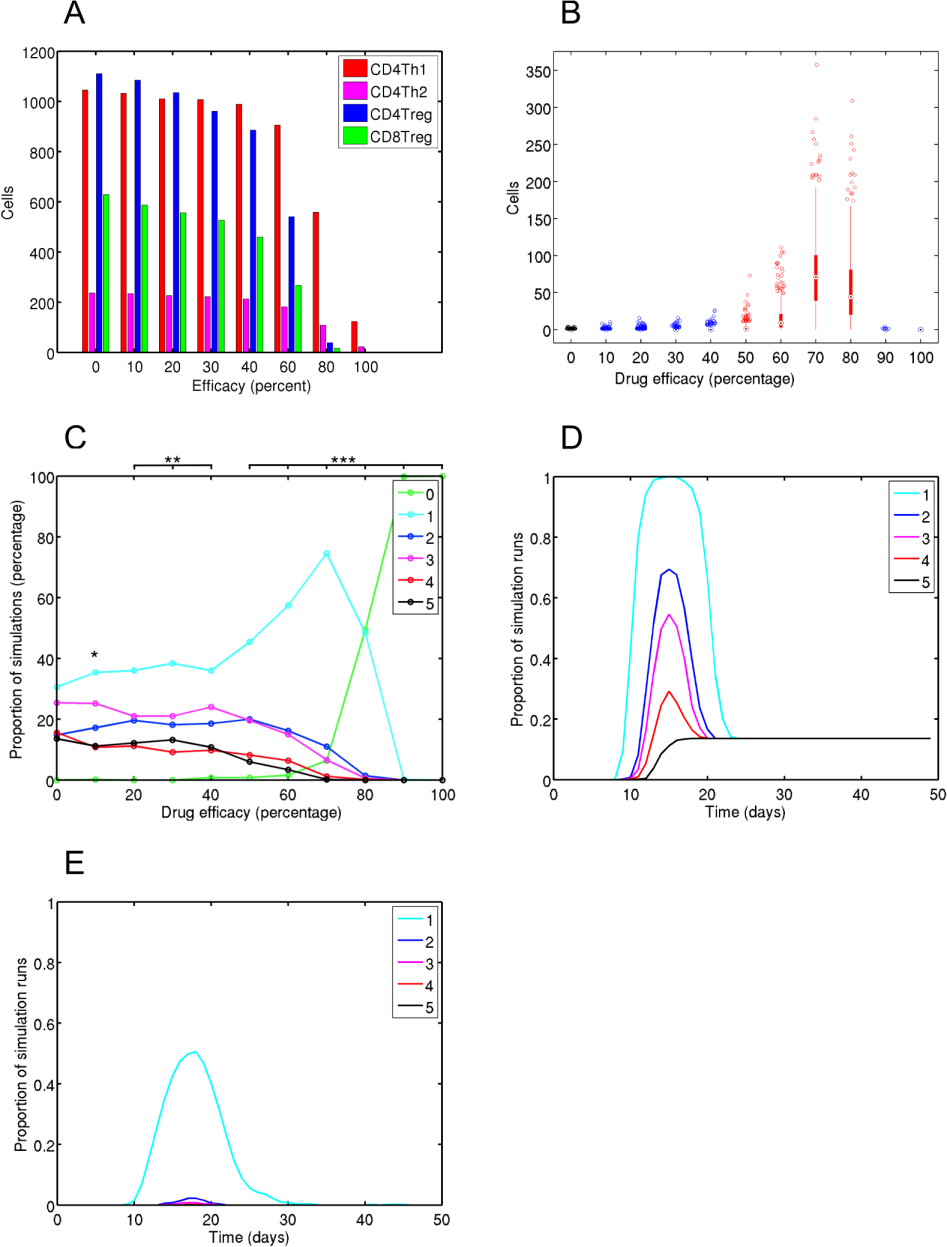
Effector T cell and clinical disease dynamics given anti-CD3 intervention at day 4. Various efficacies of anti-CD3 intervention have been administered at day 4, which corresponds with encephalitogenic T cell priming. (A) Median effector T cell peak population sizes. (B) CD4Th1 population sizes at 40 days post-induction of EAE; red and blue bars indicate large and non-large effect magnitude changes with respect to the control group, in black. (C) Proportion of simulations that reach a particular maximum clinical disease score. A-test effect magnitude levels are given: 1, 2 and 3 *'s represent small, medium and large effects respectively. (D & E) Proportion of simulations contracting particular clinical scores or greater over time, for control (D) and a drug efficacy of 80% (E).
