# Supplementary figures and images for: Determining Disease Intervention Strategies Using Spatially Resolved Simulations
Source: PLoS One. 2013 Nov 14;8(11):e80506. doi: 10.1371/journal.pone.0080506 (PMC3828403; doi:10.1371/journal.pone.0080506)

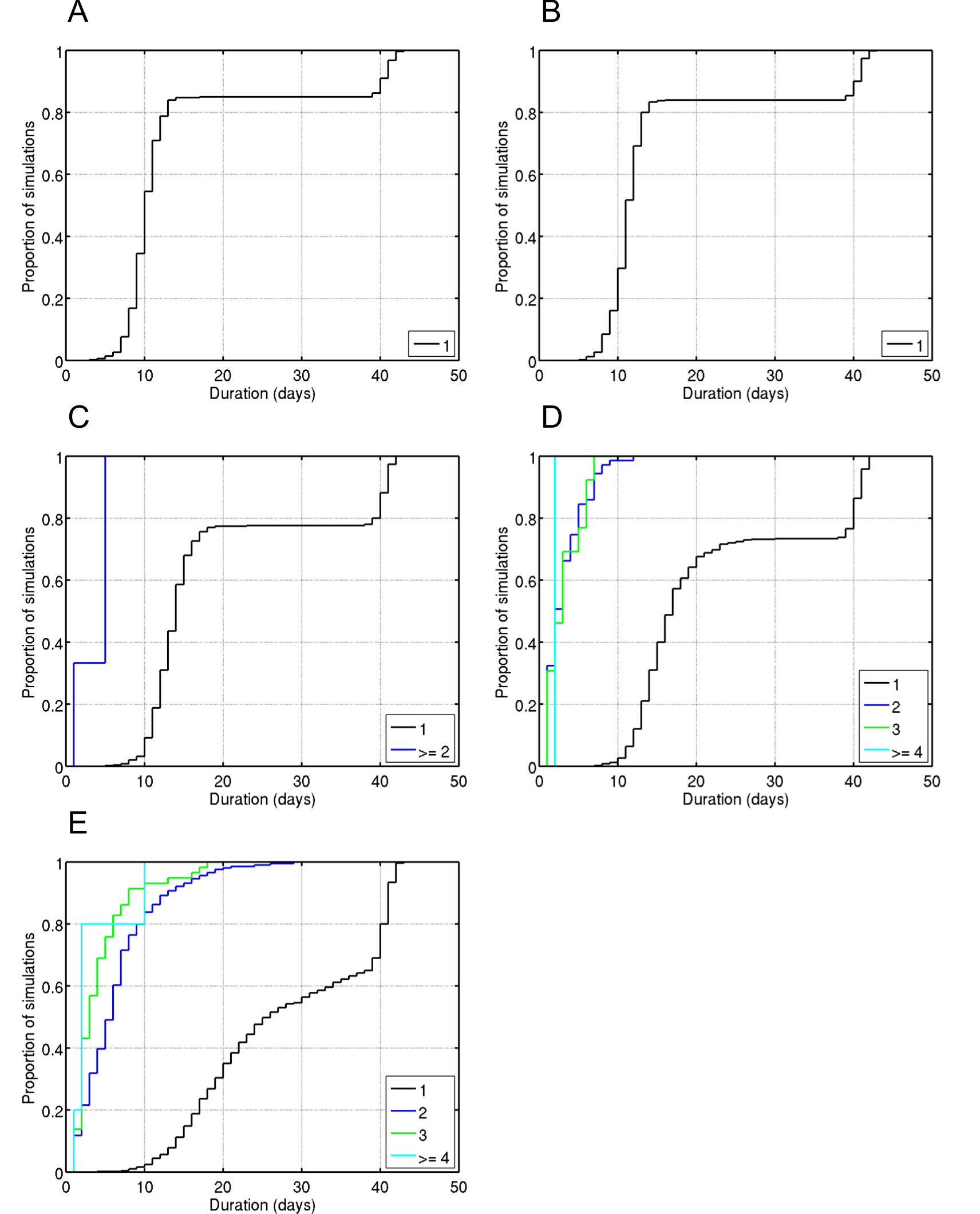

Supplement: Figure S1 — Lower regulatory efficacies increase duration of clinical episodes, and raise the mortality rate. The proportion of simulations contracting particular durations of clinical episodes or symptoms, for regulatory efficacies of 100% (A), 20% (B), 5% (C), 2% (D), 0% (E). Where applicable, data for clinical relapses are also shown. Where simulations perish, the duration of the clinical episode is from onset of symptoms until end of observation at 50 days, hence the curves plateau at the death rate, and rise to a proportion of 1 at the end. (TIF) [file pone.0080506.s001.tif]

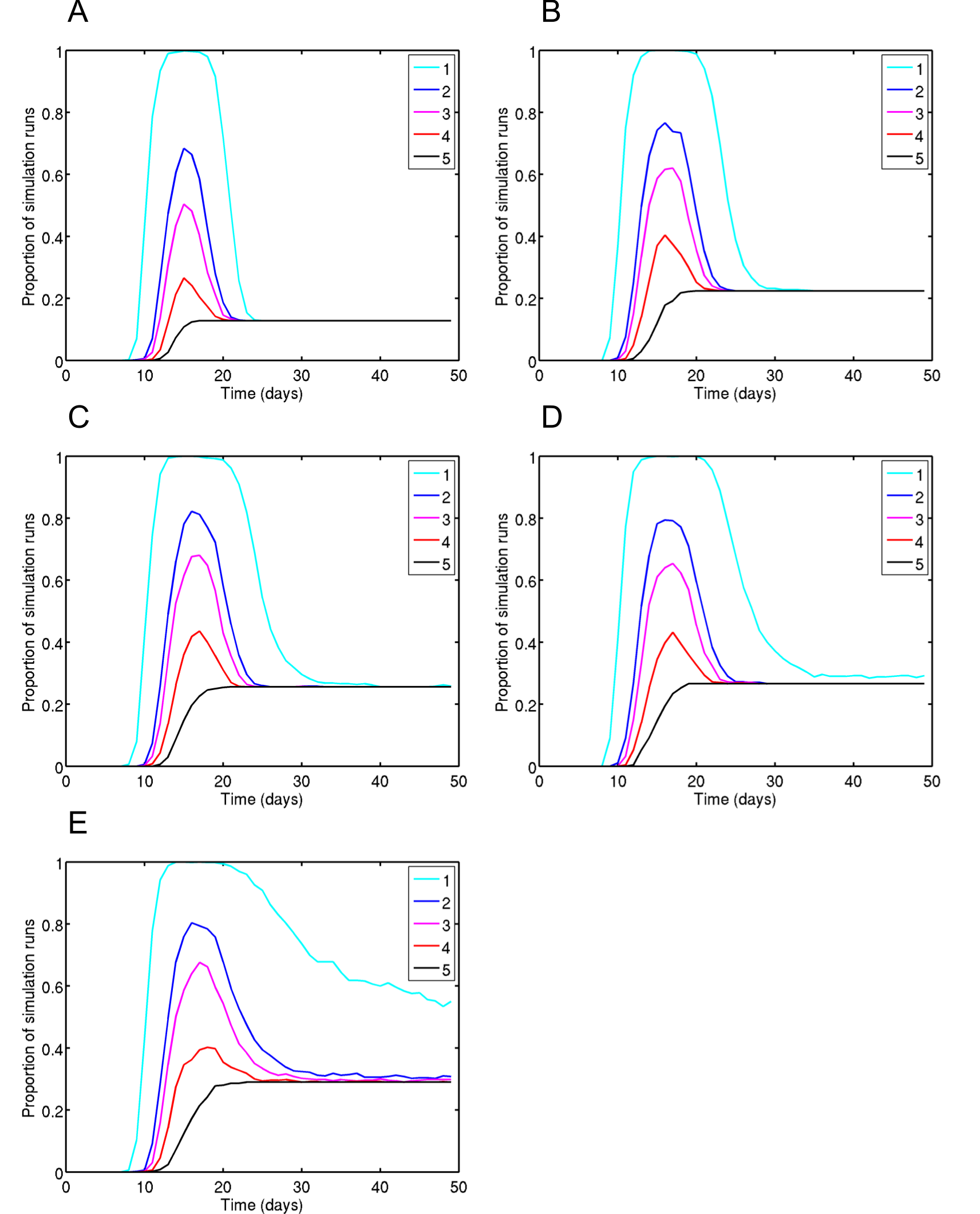

Supplement: Figure S2 — Lower regulatory efficacies increase the severity of clinical episodes. The proportions of simulations experiencing at least each level of severity of EAE over time, for regulatory efficacies of 60% (A), 5% (B), 3% (C), 2% (D), and 0% (E). (TIF) [file pone.0080506.s002.tif]

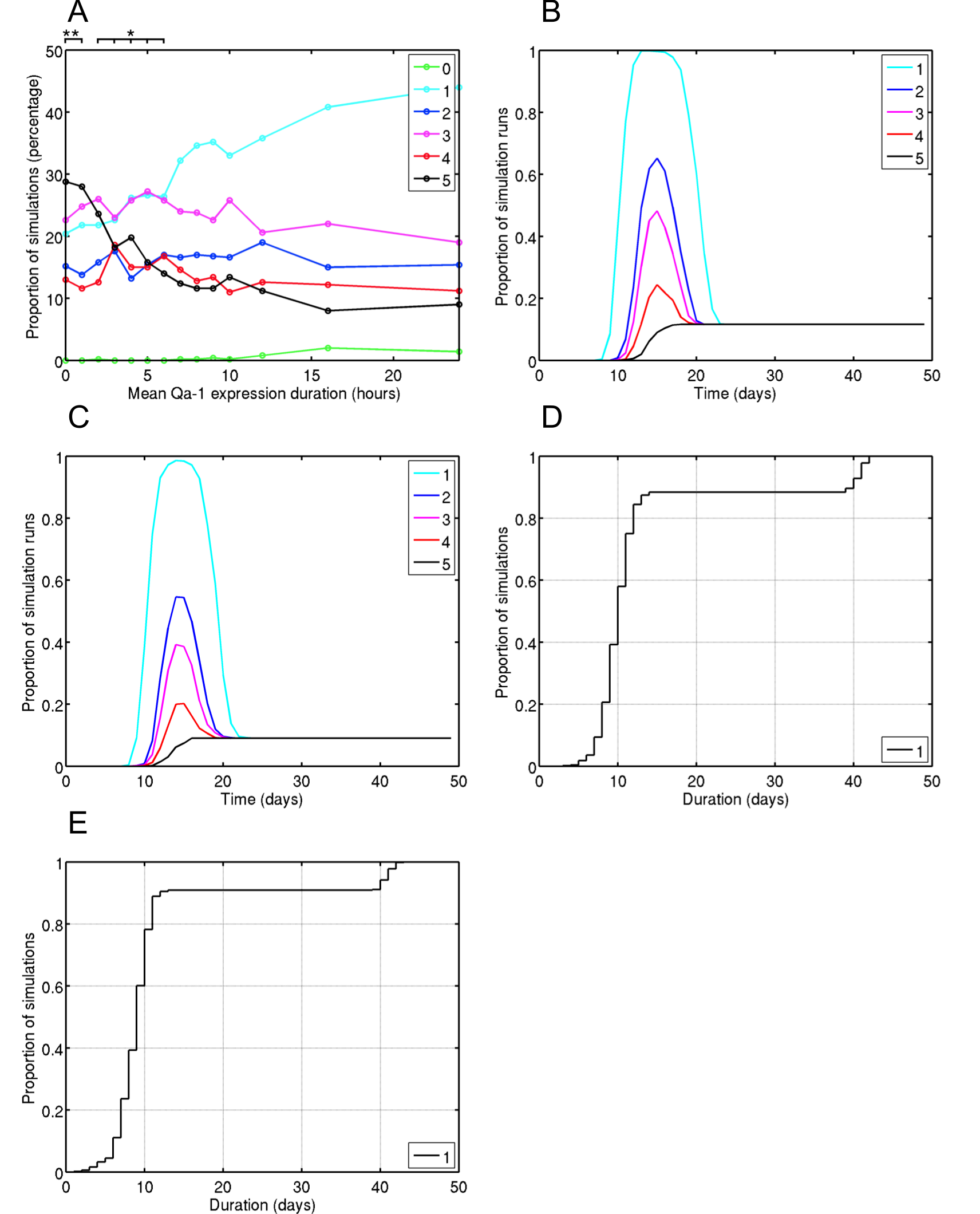

Supplement: Figure S3 — Increasing the duration of Qa-1:peptide expression beyond 8 hours has a marginal effect on clinical symptoms. The effect of altering mean duration of Qa-1:peptide complexes expression by effector CD4Th1 cells on the dynamics of clinical autoimmunity. (A) The proportions of simulations experiencing particular maximum clinical scores at any point in time. A-test effect magnitude levels are given: 1, 2 and 3 *'s represent small, medium and large effects respectively. (B & C) The proportions of simulations experiencing particular clinical scores or greater over time, for Qa-1:peptide complex expression durations of 8 hours (B) and 24 hours (C). A cumulative distribution plot showing the proportions of simulations that experience particular durations of clinical symptoms or less for Qa-1 peptide complex expression durations of 8 hours (D) and 24 hours (E). Increasing the mean period of time for which encephalitogenic CD4Th1 cells express Qa-1:peptide complexes, necessary for their regulation by CD8Treg cells, marginally reduces the severity of EAE contracted and reduces the median duration of clinical symptoms amongst simulations that do not perish from 10 days to 9. (TIF) [file pone.0080506.s003.tif]

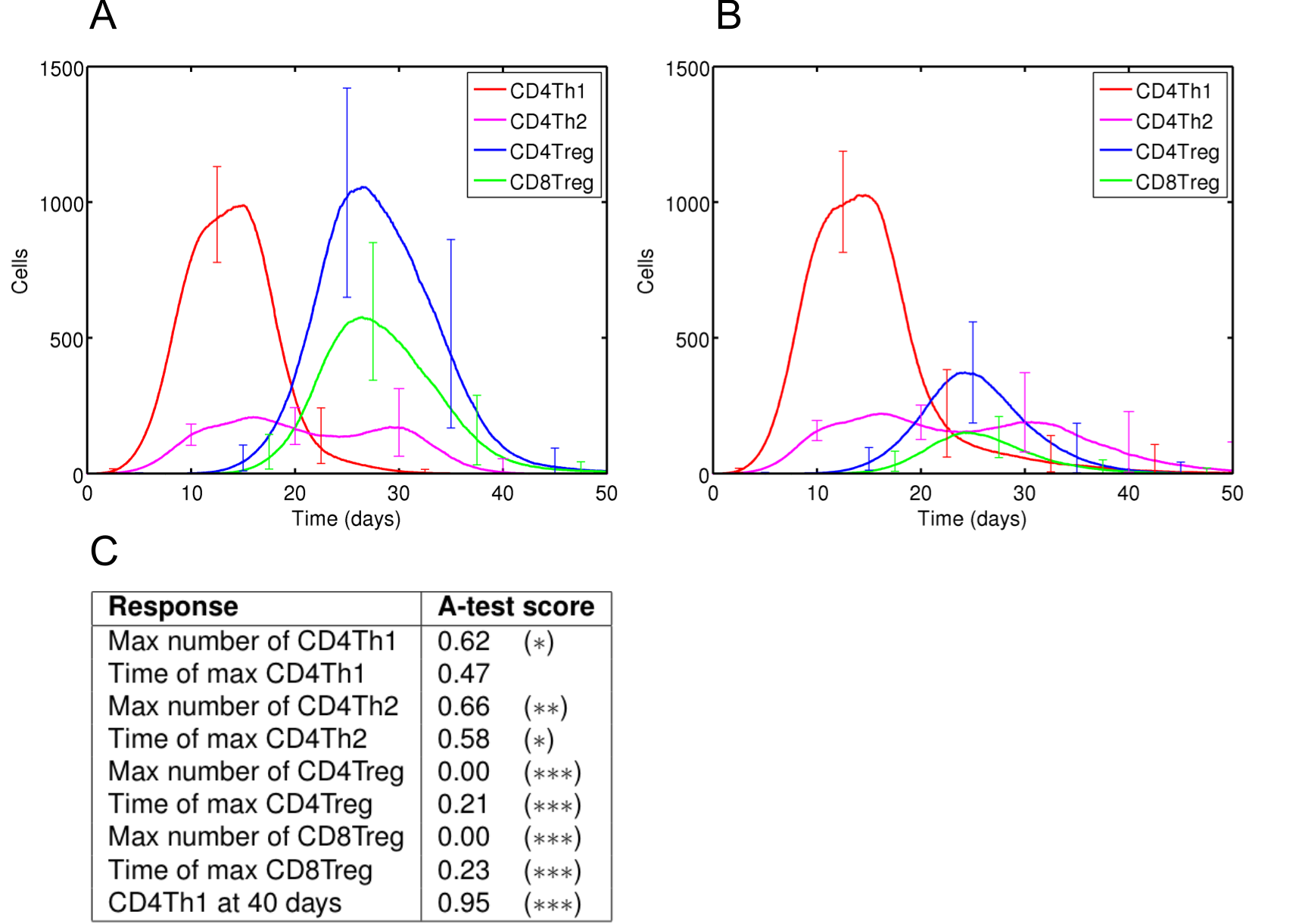

Supplement: Figure S4 — The effect of splenectomy on recovery from EAE in vivo, and on Treg priming in silico. (A & B) effector T cell dynamics in control (A) and splenectomy (B) groups. (C) statistical magnitudes effect of splenectomy on effector T cell population dynamics. The peak number of cells attained, and the times at which these occur, in each of 500 splemenctomized simulations are contrasted with similar data from a control group using the A-test. A-test effect magnitude levels are given: 1, 2 and 3 *'s represent small, medium and large effects respectively. (TIF) [file pone.0080506.s004.tif]

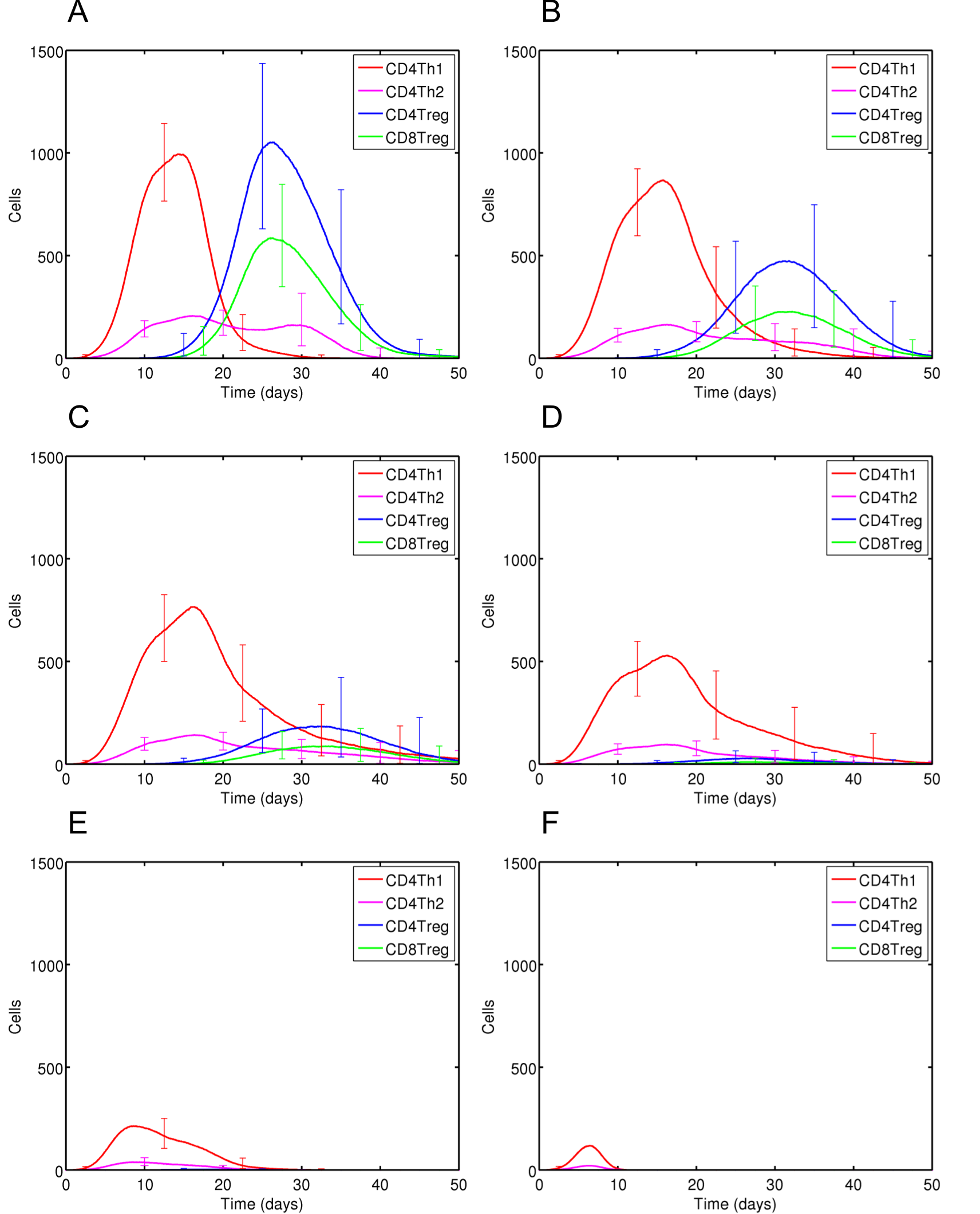

Supplement: Figure S5 — Anti-CD3 intervention at day 4 suppresses all T cell population expansions. Effector T cell population sizes over time, for anti-CD3 treatment efficacies of 0% (A), 60% (B), 70% (C), 80% (D), 90% (E), 100% (F). Higher intervention efficacies reduce effector T cell peak population sizes, but for efficacies under 80% encephalitogenic CD4Th1 cells persist for longer. (TIF) [file pone.0080506.s005.tif]

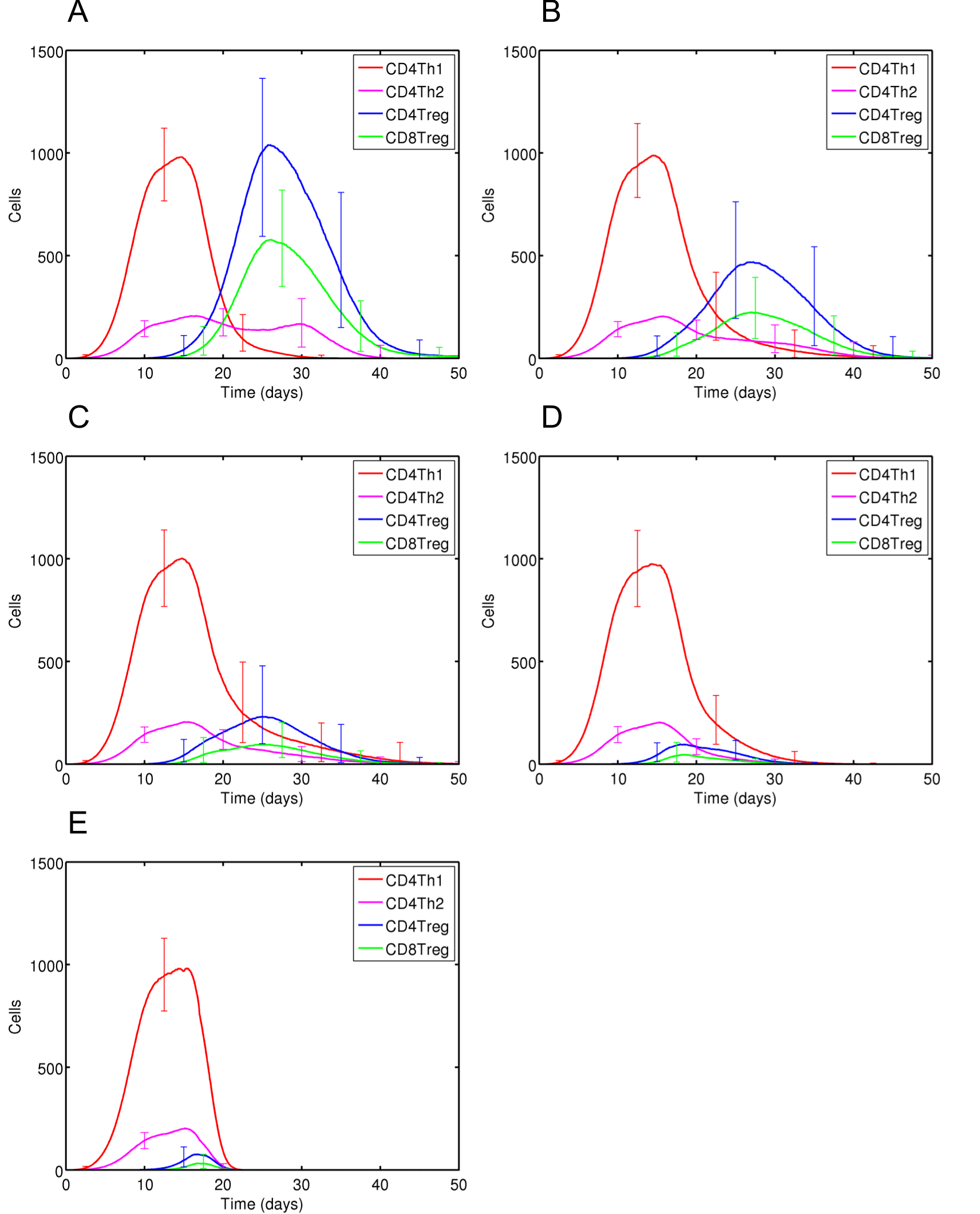

Supplement: Figure S7 — Effector T cell dynamics for various day 15 anti-CD3 intervention efficacies. Effector T cell population sizes over time, efficacies of 0% (A), 70% (B), 80% (C), 90% (D), 100% (E). (TIF) [file pone.0080506.s007.tif]

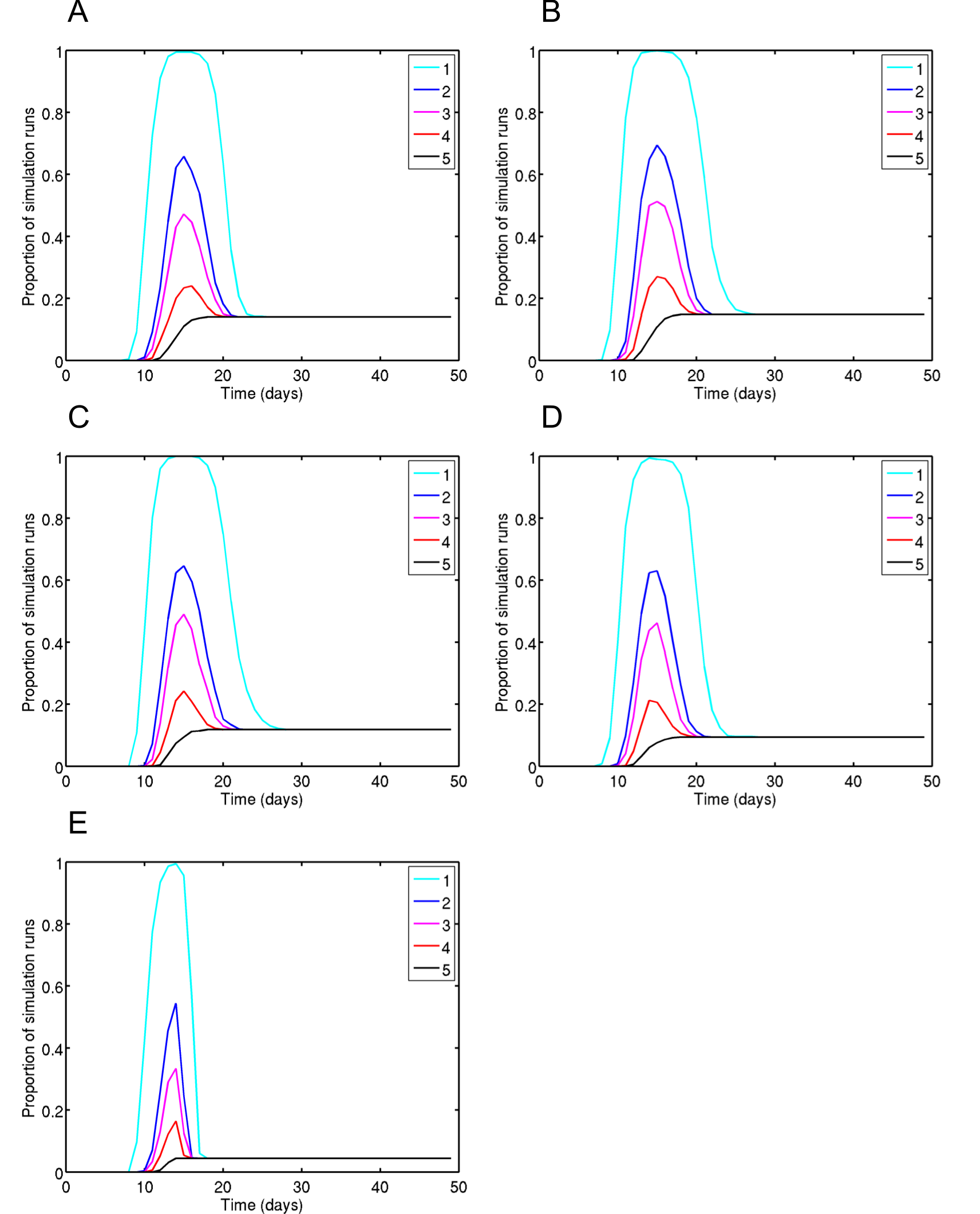

Supplement: Figure S8 — Increasing day 15 anti-CD3 efficacies reduce duration and severity of clinical episodes. Proportion of simulations experiencing particular clinical scores or greater over time, for anti-CD3 efficacies of 0% (A), 70% (B), 80% (C), 90% (D), 100% (E). (TIF) [file pone.0080506.s008.tif]

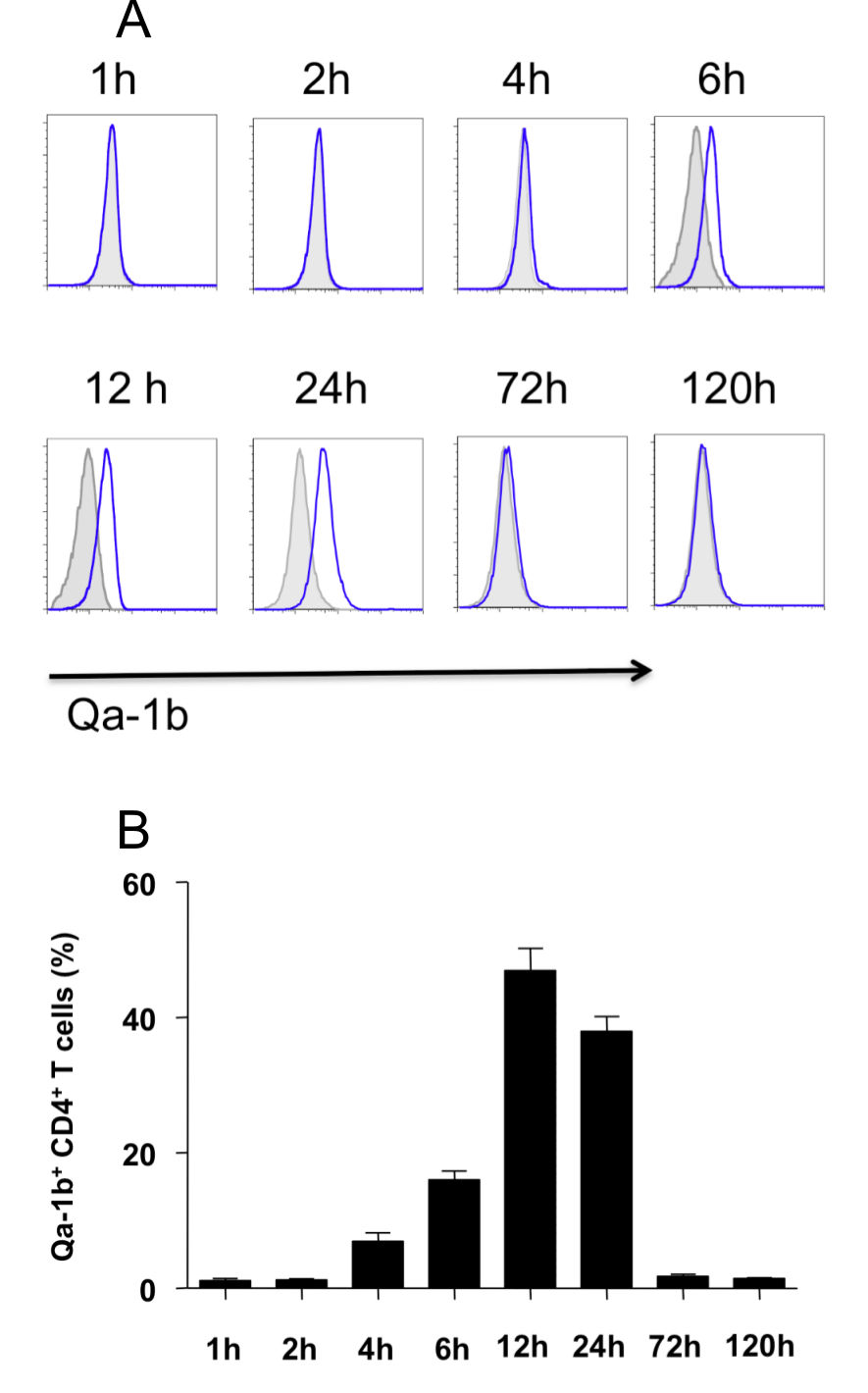

Supplement: Figure S9 — Dynamic expression of Qa-1 on T cells following their activation in vivo. Groups of C57BL/6 mice were administrated intraperitoneally with a single dose of T cell activating anti-CD3 (200 μg, 2C11) antibody. Splenocytes were harvested at indicated time points 1h, 2h, 4h, 6h, 12h, 24h, 72h, 120 h, stained with anti-TCR, anti-CD4 and anti-Qa-1b, and subjected to flow cytometry. (A) Histogram data and (B) a bar graph showing percentage of cells stained from different animals. This data is one representative of at least three individual experiments. (TIF) [file pone.0080506.s009.tif]

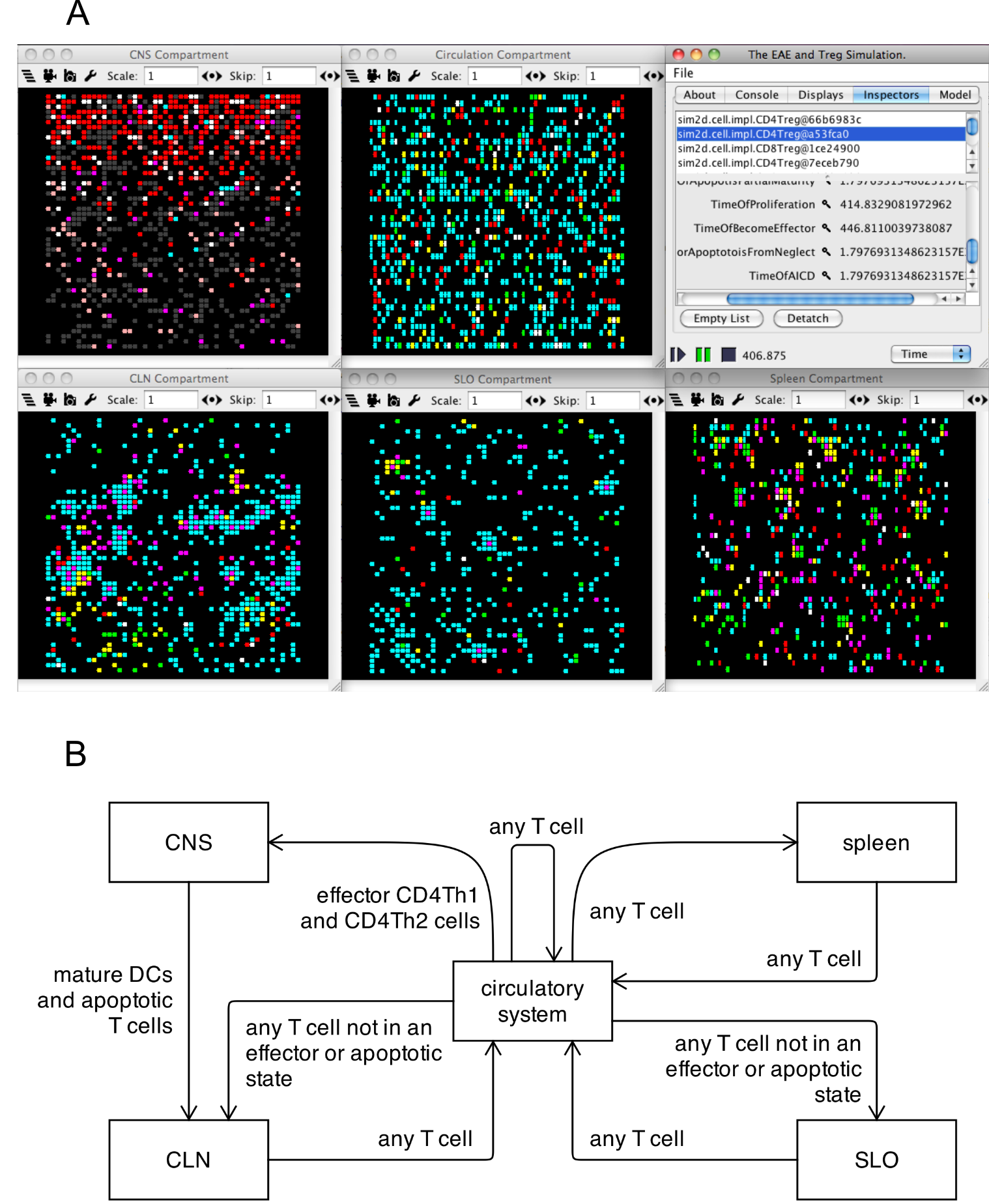

Supplement: Figure S11 — The ARTIMMUS simulation. (A) Screen shot of the simulation. Cells are coloured as follows: blue, non-effector CD4Th cells; red, effector CD4Th1 cells; white, effector CD4Th2 cells; yellow, CD4Treg cells; green, CD8Treg cells; purple, dendritic cells; peach, microglia; grey, neurons. (B) The spatial compartments represented in ARTIMMUS, and which cells are able to migrate between them. (TIF) [file pone.0080506.s011.tif]

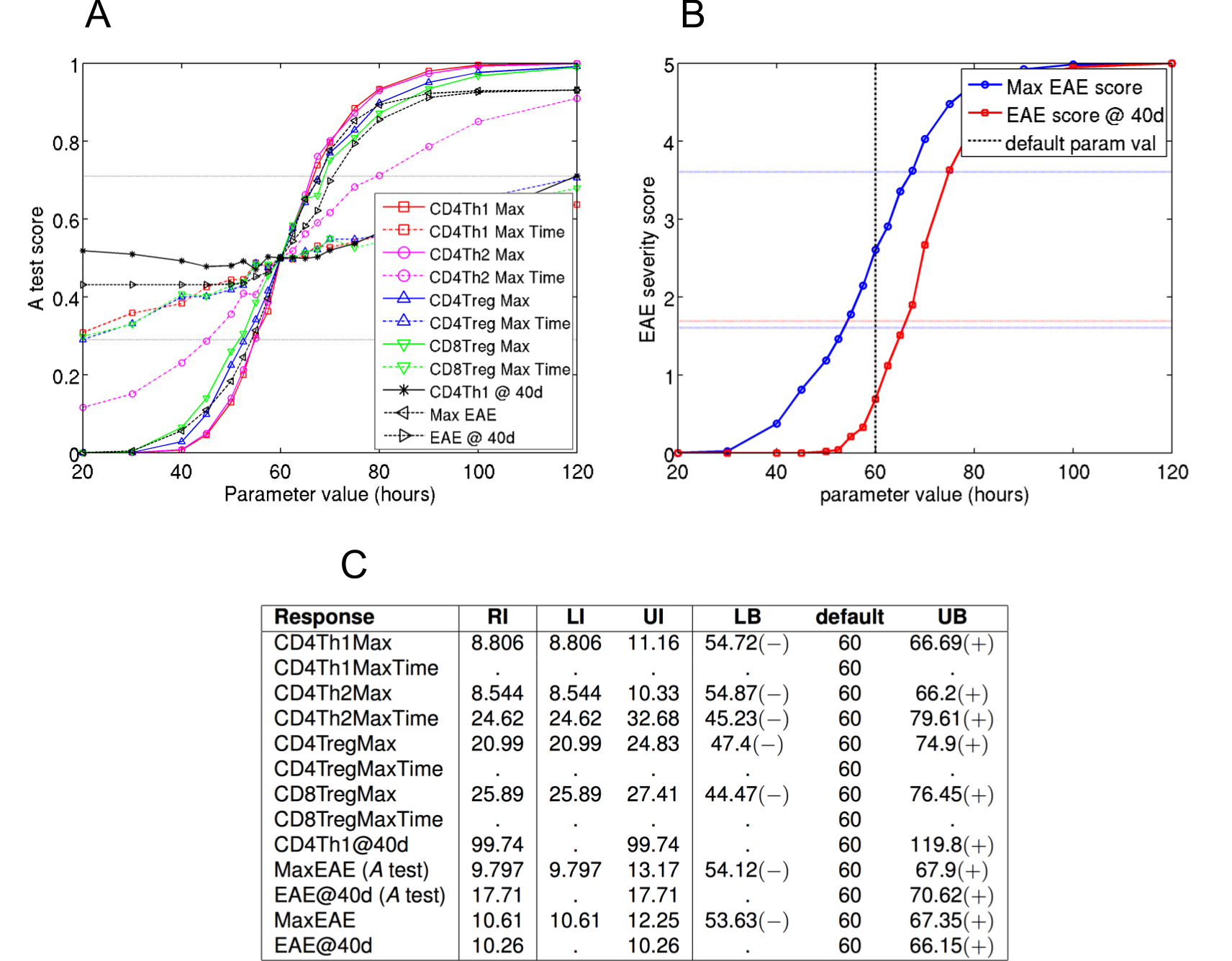

Supplement: Figure S15 — Example robustness analysis of the TCell_AICDMean parameter. This parameter dictates the mean lifespan of effector T cells before they apoptose due to activation induced cell death (AICD). The analysis establishes the range of values that this parameter may take before significant deviations in various aspects of simulation behaviour take place. These aspects, termed responses, are as follows: the …Max and …MaxTime responses indicate the peak population size for each T cell population and the times at which this peak occurred. CD4Th1@40d represents the CD4Th1 population size at 40 days. Max EAE and EAE@40 represent the disease severity score at its peak and at 40 days; these measures are tested for significant deviation through both the A-test and ±1.0 of the default value. (A) A-test scores indicating how changes parametric perturbation influences simulation responses, the ‘large’ effect magnitude boundaries are indicated. (B) Change in EAE scores under parametric perturbation, ±1.0 boundaries are indicated. (C) Summary of robustness indices, lower and upper boundaries and indices for all response. RI, robustness index; LI, lower index; UI, upper index; LB, lower boundary; UB, upper boundary. For clarity NaN (not a number) is indicated by a period. (TIF) [file pone.0080506.s015.tif]
